# A learning-evoked slow-oscillatory architecture paces population activity for offline reactivation across the human medial temporal lobe

**DOI:** 10.1016/j.neuron.2026.05.004

**Published:** 2026-06-01

**Authors:** Adrien A. Causse, Jonathan Curot, Vítor Lopes-dos-Santos, Raphaël Nunes-da-Silva, Helen C. Barron, Vincent Dornier, Marie Denuelle, Amaury De Barros, Jean-Christophe Sol, Jean-Albert Lotterie, Katia Lehongre, Sara Fernandez-Vidal, Valerio Frazzini, Vincent Navarro, Luc Valton, Emmanuel J. Barbeau, Tim Denison, Leila Reddy, David Dupret

**Affiliations:** 1 Brain Network Dynamics Unit, Nuffield Department of Clinical Neurosciences, University of Oxford, Oxford, UK; 2 Medical Research Council Centre of Research Excellence in Restorative Neural Dynamics, Oxford, UK; 3 CerCo, CNRS UMR5549, University of Toulouse, Toulouse, France; 4 Brain Electrophysiology, Epilepsy and Sleep Unit, Neurology Department, Toulouse University Hospital, Toulouse, France; 5 Oxford Centre for Integrative Neuroimaging, University of Oxford, FMRIB, John Radcliffe Hospital, Oxford, UK; 6 Department of Neurology and Neurosurgery, Toulouse University Hospital, Toulouse, France; 7 Toulouse Neuro Imaging Center, INSERM, U1214, Toulouse, France; 8 Centre de Neuro-Imagerie de Recherche, ICM Paris Brain Institute, Pitié-Salpêtrière Hospital, Paris, France; 9 Sorbonne Université, Paris Brain Institute, ICM, Inserm, CNRS, Pitié-Salpêtrière Hospital, Paris, France; 10 Assistance Publique-Hôpitaux de Paris, Epilepsy and EEG Units and Reference Center of Rare Epilepsies, ERN EpiCare, Pitié-Salpêtrière Hospital, Paris, France

## Abstract

Memory processing requires coordinated engagement of neuronal populations across brain networks and over time. How such coordination is organized in the human medial temporal lobe (MTL) remains unclear. Here, we show that MTL population activity is dynamically structured by a transient slow-oscillatory architecture that emerges during learning to promote offline consolidation and later recall. Using intracranial recordings that combine single-neuron spiking activity and local field potentials in human participants, we find that mnemonic engagement elicits on-demand slow-oscillatory bursts in the hippocampus. These hippocampal bursts synchronize gamma-band patterns across MTL regions, defining discrete coordination events that pace cross-regional coactivity motifs during learning. These learning-evoked population motifs are selectively reactivated during hippocampal ripples in post-learning rest, and the strength of their reactivation predicts subsequent recall accuracy. Together, these findings identify a multi-scale coordination mechanism that links distributed population activity across learning, consolidation, and recall in humans.

## Graphical abstract

**Graphical abstract:**
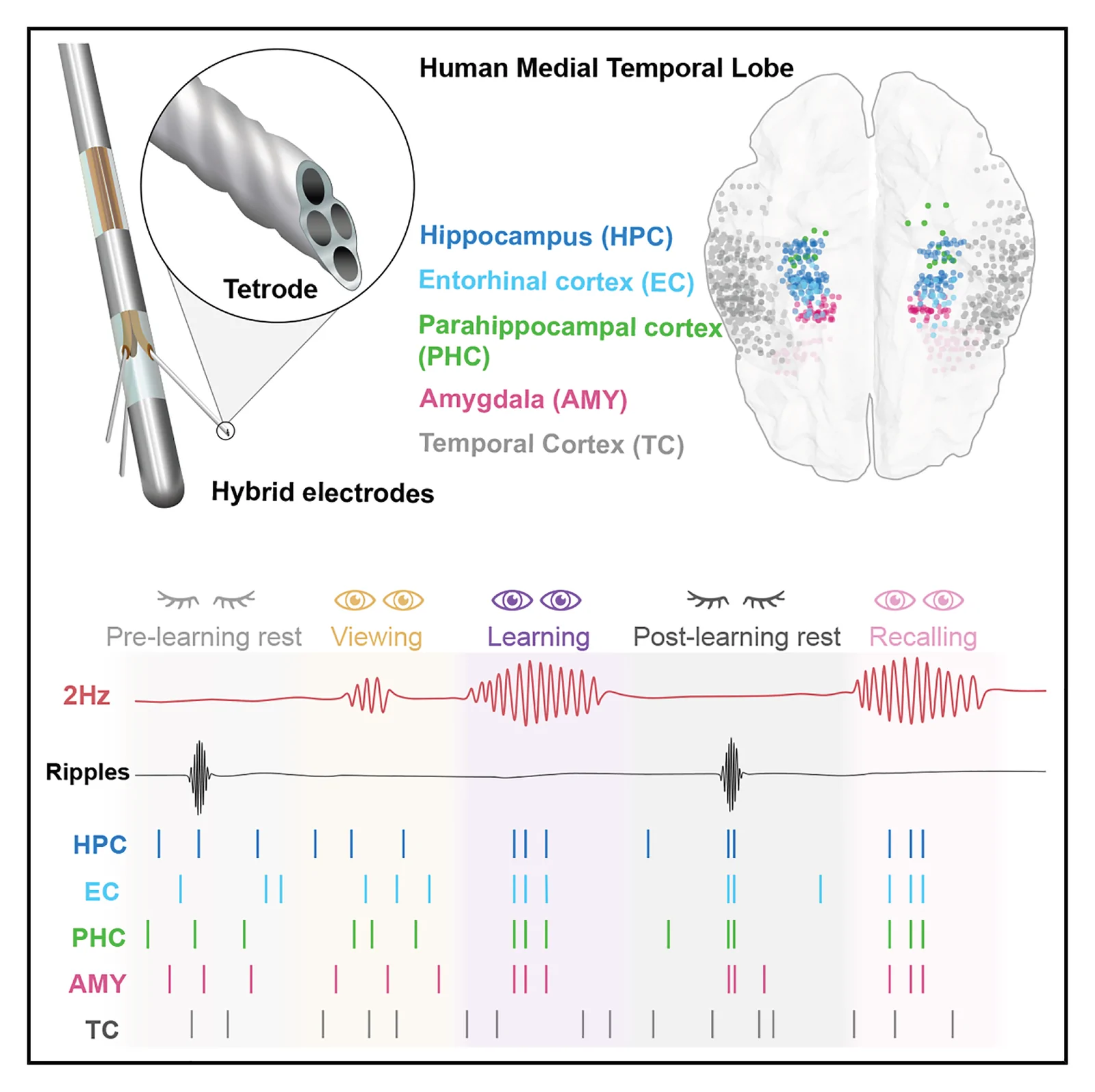

## Introduction

Memory unfolds across an extended processing arc that spans experience and time, from learning through consolidation to recall.^1–3^ At the core of the brain-memory circuitry is the hippocampus, whose neurons support the representation of relationships between stimuli and events.^4–8^ During learning, hippocampal activity gives rise to structured patterns of neuronal coactivity, which are subsequently reactivated offline during rest and later reinstated online during recall.^9–11^ Although these processes have been extensively characterized at the behavioral and representational levels, the population-level network mechanisms that dynamically coordinate hippocampal activity across learning, consolidation, and recall in humans remain unclear.

Coordinating memory across this processing arc requires interactions between the hippocampus and the broader medial temporal lobe (MTL) network.^12,13^ The hippocampus interacts with entorhinal, parahippocampal, and amygdala regions to support memory. Integrating activity across this multi-region system poses a fundamental challenge. Memory-related processing requires integration of neuronal spiking, population-level synchronization, and interregional communication across multiple temporal and spatial scales, while preserving local computational dynamics within individual regions.^14^ Achieving such coordination demands mechanisms capable of transiently binding distributed neural activity into coherent functional states and organizing population-level communication across regions. Oscillatory activity provides a powerful means of achieving this coordination by defining temporal windows in which patterns of neuronal coactivity form, interact, and become eligible for subsequent offline consolidation and later reinstatement.

In animal models, hippocampal oscillations have been shown to provide a temporal framework for coordinating memory-related population activity.^15^ In rodents, theta-band (5–12 Hz) oscillations are readily observed as rhythmic fluctuations in local field potentials (LFPs) during voluntary movement and spatial exploration.^16,17^ These oscillations provide a temporal scaffold for organizing millisecond-timescale patterns of neuronal coactivity during learning and their reinstatement during memory-guided behavior.^18–21^ Theta-nested gamma-band oscillations (30–150 Hz) have been proposed to reflect both local population computation within the hippocampus and communication channels through synchronization with distributed brain regions.^22–25^ Patterns of neuronal coactivity formed during theta-governed online states are subsequently reactivated during high-frequency ripple events (100–250 Hz) that occur during rest and sleep, supporting offline memory consolidation.^9,19,20,26–30^

Together, these findings have shaped influential models of memory and cognition in which oscillatory dynamics link online processing during awake behavior with subsequent consolidation during offline states.^7,10,31–34^ However, accumulating evidence indicates that hippocampal oscillatory dynamics vary substantially across species and behavioral contexts. In larger-brained mammals, including rabbits, cats, bats, and primates, hippocampal rhythmic activity is often slower, more intermittent, and more closely linked to task demands than to behavioral exploration.^35–38^ In humans, intracranial recordings frequently indicate lower-frequency activity whose functional significance remains elusive.^39–43^ As a result, it is unclear whether the human MTL expresses a unifying coordination architecture capable of linking neuronal spiking, network synchronization, and offline reactivation across memory processing stages.

Here, we show that human memory is organized by a coordination architecture in which brief, task-evoked rhythmic events link neuronal spiking, interregional synchronization, and offline reactivation across the full memory processing arc. Using intracranial electroencephalography combined with simultaneous single-neuron recordings from the human MTL during a relational memory task, we identify a coordination architecture expressed as transient bouts of task-evoked 2-Hz oscillatory activity during learning and recall. These slow-oscillatory “burst” events pace neuronal population spiking and synchronize gamma-band activity patterns across hippocampal and extra-hippocampal MTL regions, defining temporal windows for coordinated network interactions. Coactivity motifs structured by this oscillatory architecture during learning are selectively reactivated during hippocampal ripple events in post-learning rest. The strength of this reactivation predicts subsequent recall accuracy, linking oscillatory coordination during learning to offline consolidation and later memory performance. Together, these findings reveal an oscillatory architecture that operates through discrete, mnemonic engagement-locked burst events rather than as a sustained background rhythm, coordinating MTL activity across learning, consolidation, and recall in humans.

## Results

### Mnemonic engagement elicits transient slow-oscillatory bursts in the human hippocampus

**Figure 1:**
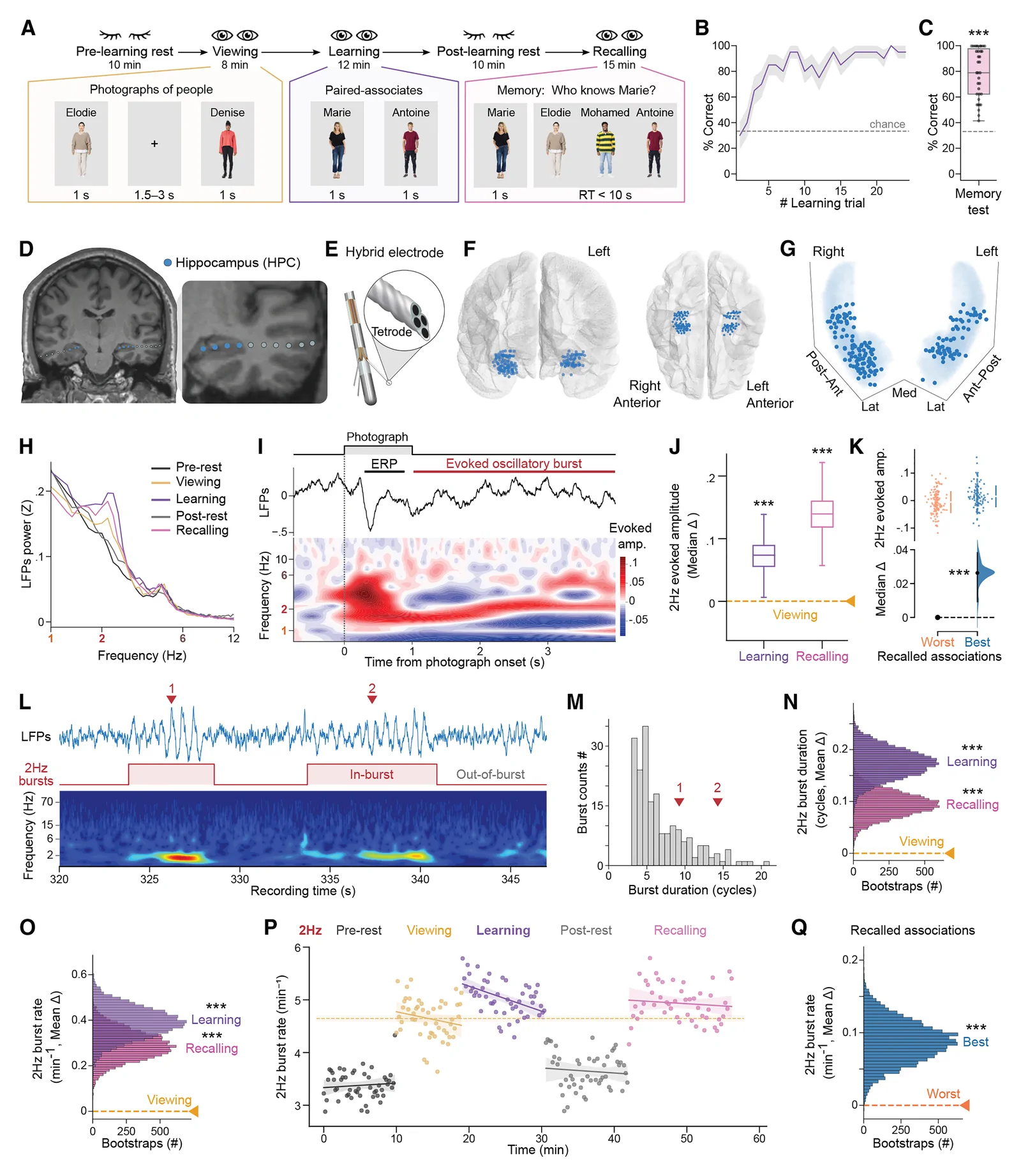
Mnemonic engagement evokes slow-oscillatory bursts in the human hippocampus (A) Schematic of the relational memory task with example stimulus photographs. (B) Learning performance (percentage correct trials; mean ± SEM). (C) Memory recall accuracy (percentage correct responses). The box indicates the interquartile range (IQR), the center line indicates the median, and the whiskers extend to 1.5× IQR. (D) T_1_-weighted MRI showing contact locations from two representative depth electrodes targeting the hippocampus. (E) Hybrid micro-/macro-electrode design with tetrodes extending from the macrocontact shaft. (F and G) Montreal Neurological Institute (MNI) brain template (F) and 3D projection (G) of hippocampal contacts across participants (axes: Post-Ant, posterior-anterior; Med-Lat, medio-lateral). (H) Power spectral density (PSD) from an example hippocampal contact across task stages, illustrating task-dependent modulation of slow oscillatory activity. (I) Peri-stimulus average of hippocampal LFPs (top) and corresponding spectrogram (bottom), showing a stimulus-locked event-related potential (ERP) followed by transient slow oscillatory activity during the inter-stimulus interval. (J) Median differences in evoked 2-Hz oscillatory amplitude during learning and recall relative to viewing, computed over post-ERP epochs (>1 s after photograph onset; whiskers extend to 95% CIs). Note that the two photographs presented during learning were contiguous (directly linked as paired associates), which may have interrupted evoked 2-Hz oscillations (e.g., through phase reset^44^). This was not the case during the recall session (see also Figure S1P and STAR Methods). (K) Estimation plot showing the differences in amplitude of evoked 2-Hz oscillatory bursts during learning trials associated with best versus worst subsequent memory recall. Upper: raw data (points) with mean ± SD (vertical lines); bottom: mean difference (black dot) with 95% CI (black ticks) and bootstrapped sampling-error distribution (filled curve) relative to the worst-recalled associations reference (horizontal dashed line). (L and M) Hippocampal slow-oscillatory bursts. (L) Example raw LFP trace (top) showing 2-Hz oscillatory bursts with corresponding spectrogram (bottom). (M) Distribution of burst durations for the contact shown in (L); arrowheads indicate durations of the two bursts visible in (L). (N and O) Bootstrapped mean differences in 2-Hz burst duration (N) and rate (O) during learning and recall relative to viewing. (P) Time course showing expression dynamics of hippocampal 2-Hz burst rate in the relational memory task, averaged across participants and contacts. Lines indicate linear fits, and shaded areas 95% CIs. Burst rate was negatively correlated with time as learning progressed (learning, *r* = −0.48, *p* < 0.001; other task sessions, *r* > −0.20, *p* > 0.161). (Q) Bootstrapped mean differences in 2-Hz burst rate during learning trials associated with best versus worst subsequent memory recall. Data were analyzed using two-sided paired permutation tests, except in (C), where a Wilcoxon signed-rank test was applied; ***p < 0.001.

To examine how hippocampal network dynamics evolve across the memory processing arc in humans, we designed an associative relational task that enabled continuous intracranial recordings from learning through consolidation to recall (Figure 1A). In this task, we trained 27 participants to learn associations between individuals in a community. Each recording day began with a prelearning rest (pre-rest), followed by a viewing session during which participants were familiarized with photographs of community members presented in random order. During the subsequent learning session, participants learned associations between pairs of individuals (for example, “*Marie knows Antoine*”). Learning progress was assessed through intermittent multiple-choice questions confirming knowledge of the community structure (Figure 1B). After a post-learning rest (post-rest), participants completed a memory recall test session that confirmed successful retention of the learned associations (Figure 1C; *p* < 0.001; Wilcoxon signed-rank test compared with the 33% chance level).

Intracranial recordings were obtained using hybrid depth electrodes that enabled simultaneous measurement of hippocampal LFPs and population-level single-neuron spiking (Figures 1D–1G and S1A–S1C). Each electrode shaft incorporated multichannel tetrodes (Figure 1E), similar to those commonly employed in rodent studies,^25,45^ combined with macrocontacts for clinical epilepsy monitoring.^46^ To evaluate the network expression of a coordination architecture capable of linking hippocampal activity across memory stages, we decomposed LFP signals into their constituent oscillatory components using an unsupervised, data-driven approach that does not impose predefined frequency bands, previously validated in rodents (Figures S1C–S1F).^20,25,45^ Using this approach, we identified a prominent slow-oscillatory component centered near 2 Hz (peak [80% power band]: 2.38 [1.25–3.50] Hz), alongside a slower (~1 Hz) and a faster (~6 Hz) rhythmic component (Figures S1C–S1F). Notably, standard local (bipolar) referencing markedly reduced sensitivity to this slow component (Figures S1G–S1I), and its expression was strongest at electrode contacts outside interictal zones (Figures S1J and S1K),^47^ providing a potential explanation for why it has been difficult to detect in prior human studies.

Mnemonic engagement—here defined as task periods involving active acquisition or retrieval of associations rather than passive viewing—was associated with enhanced expression of this slow-oscillatory component in the hippocampus (Figures 1H and 1I). Hippocampal 2-Hz power was significantly greater during both learning and recall than during resting or viewing sessions (learning, *p* < 0.001; recalling, *p* = 0.006; paired permutation tests compared with pre-learning rest), with no comparable modulation observed at adjacent slower or faster frequencies (Figures S1L and S1M). Linear mixed-effects modeling confirmed that this enhancement was selective for the 2-Hz component (Figure S1N). Peri-stimulus averages of hippocampal LFPs revealed a photographic stimulus-locked event-related potential (ERP), with slow oscillatory activity emerging during the inter-stimulus interval (Figures 1I and S1O). The amplitude of this 2-Hz activity was significantly enhanced during learning and recall (Figures 1J and S1P; learning, *p* < 0.001; recalling, *p* < 0.001; paired permutation tests compared with viewing), and correlated with the magnitude of the ERP deflection (Figure S1Q), with no comparable modulation for neighboring 1- or 6-Hz bands (Figures S1Q and S1R). Photographs that elicited the strongest 2-Hz response amplitude during learning were those subsequently best remembered (Figure 1K; best versus worst recalled associations, *p* < 0.001; paired permutation tests).

This slow oscillatory activity was expressed as brief, stimulus-locked bursts during the task (mean maximal burst duration [95% confidence interval (CI)]: 19.6 [15.8–23.4] cycles per burst; Figures 1L, 1M, and S1S), rather than as sustained rhythmic activity. The rate and duration of hippocampal 2-Hz bursts increased during learning and recall relative to viewing (Figures 1N and 1O). The rate and duration of 2-Hz bursts were higher at the beginning of learning and decreased as learning progressed (Figures 1P and S1T). This expression profile was not observed for higher-frequency (6-Hz) bursts (Figures S1U–S1X). Photographs best remembered during recall were associated with higher 2-Hz burst occurrence during learning (Figure 1Q). Higher task performers showed greater 2-Hz burst rate and longer burst duration than lower task performers (Figures S1Y and S1Z).

Mnemonic engagement was therefore associated with a slow-oscillatory response in the human hippocampus expressed as stimulus-locked 2-Hz bursts. This transient activity was most prominent during acquisition of new associations, decreased as learning progressed, and predicted subsequent recall performance.

### Learning-evoked slow-oscillatory architecture paces hippocampal population spiking and gamma-band activity

**Figure 2:**
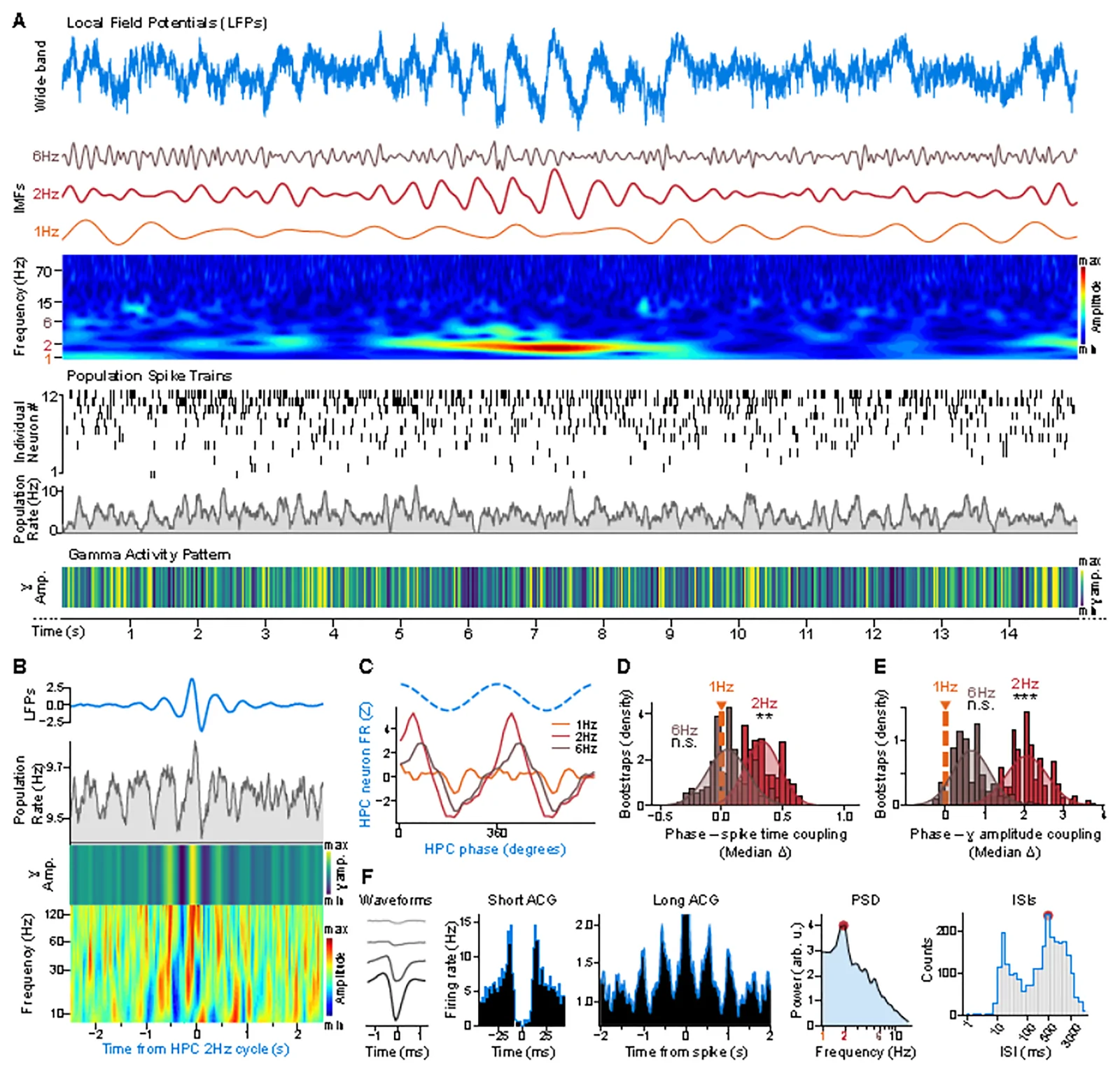
A slow-oscillatory architecture paces hippocampal spiking and gamma activity (A) Example hippocampal tetrode recording illustrating slow oscillatory activity, neuronal spiking, and gamma-band activity. From top to bottom: wideband LFP trace decomposed into its constituent oscillatory components (intrinsic mode functions [IMFs]), with spectrogram; raster plot of single-neuron spike trains with population firing rate; and corresponding gamma-band activity pattern. (B) Average hippocampal LFPs aligned to 2-Hz oscillatory phase with instantaneous population firing rate, gamma amplitude, and spectrogram, showing rhythmic modulation. (C) Firing-phase histograms of an example neuron relative to 1-, 2-, and 6-Hz oscillations. (D) Spike-phase consistency differences between 1-Hz and 2- or 6-Hz oscillations across hippocampal neurons. (E) Phase-amplitude coupling differences for hippocampal gamma envelopes between 1-Hz and 2- or 6-Hz oscillations. (F) Representative hippocampal neuron exhibiting slow-oscillatory rhythmicity. From left to right: mean spike waveform across tetrode channels, short- and long-timescale spike autocorrelograms (ACGs), power spectral density (PSD), and interspike interval (ISI) distribution. Statistics were assessed using two-sided paired permutation tests; ***p < 0.001, **p < 0.01; n.s., not significant.

To characterize the neural architecture underlying memory-associated slow oscillatory activity, we examined how hippocampal neuronal spiking and local network dynamics relate to the phase of this oscillatory signal (Figure 2A). At the population level, hippocampal spiking activity exhibited clear modulation by slow-oscillatory phase. Average hippocampal LFPs aligned to oscillatory phase revealed rhythmic modulation of population firing rates at the slow-oscillatory timescale (Figures 2B and S2A–S1C). Across neurons, spikes were more strongly phase-locked to the slow-oscillatory rhythm (2-Hz) than to slower (1-Hz) or faster (6-Hz) comparison frequencies (Figures 2C and 2D; 2-Hz versus 1-Hz, *p* = 0.003; 2-Hz versus 6-Hz, *p* = 0.020; 1-Hz versus 6-Hz, *p* = 0.815; two-sided paired permutation tests), indicating selective coordination of spiking by the slow-oscillatory signal.

Gamma-frequency activity is thought to reflect local population spiking subspaces in animal models.^24^ Consistent with this, human hippocampal spiking correlated more strongly with local than with distal gamma-band (60–160 Hz) activity (Figure S2D). The amplitude of hippocampal gamma oscillations was more strongly modulated by slow-oscillatory phase than by the phase of slower (1-Hz) or faster (6-Hz) rhythms (Figures 2B and 2E; 2-Hz versus 1-Hz, *p* < 0.001; 2-Hz versus 6-Hz, *p* < 0.001; 1-Hz versus 6-Hz, *p* = 0.097; two-sided paired permutation tests), indicating selective cross-frequency coupling. This modulation extended along the hippocampal anteroposterior axis (Figures S2E–S2K).

At the single-neuron level, hippocampal neurons also exhibited prominent slow-timescale rhythmicity in their spike train autocorrelograms (Figures 2F, S2L, and S2M), observed in both putative principal cells and interneurons (Figures S2N–S2P), confirming that slow-oscillatory structure was present at population and single-neuron scales.

Together, these findings show that learning-evoked slow oscillatory activity in the human hippocampus constitutes a coherent temporal architecture that paces population spiking and organizes local gamma-band network dynamics.

### The hippocampal slow-oscillatory architecture synchronizes activity across the MTL

**Figure 3:**
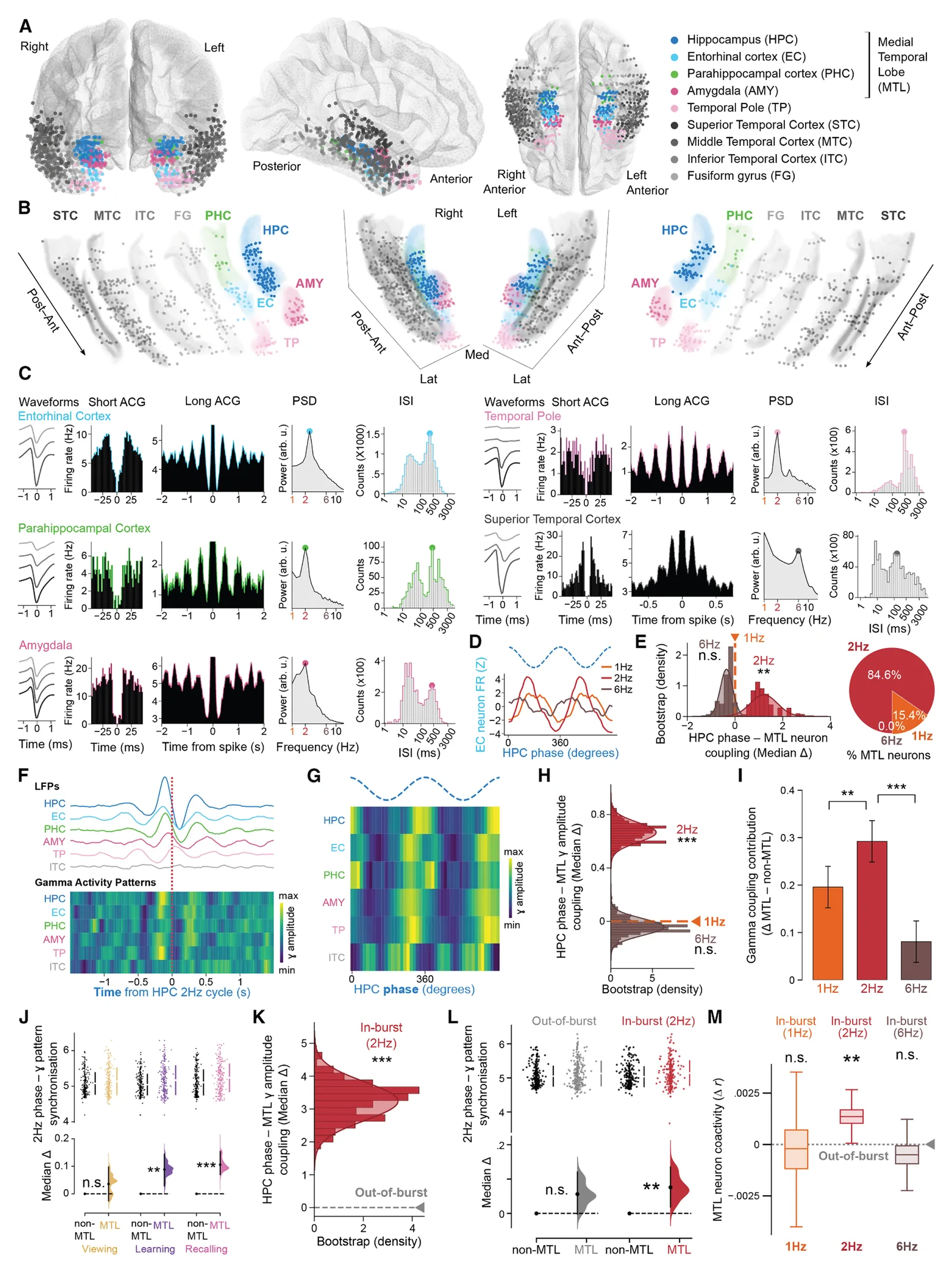
The hippocampal slow-oscillatory architecture synchronizes activity across the MTL (A and B) MNI brain template (A) and 3D projection (B; with expanded view) showing electrode contact locations across temporal lobe (MTL and non-MTL) regions in all participants. (C) Representative neurons recorded from MTL tetrodes and exhibiting 2-Hz oscillatory rhythmicity, contrasted with a non-MTL neuron exhibiting faster (6-Hz) rhythmicity. (D) Firing-phase histograms of an example entorhinal cortex neuron relative to 1-, 2-, and 6-Hz hippocampal oscillations. (E) Median differences in spike-phase consistency between hippocampal 1-Hz and 2- or 6-Hz rhythms for extra-hippocampal MTL neurons and proportion of neurons preferentially coupled to each frequency. (F) Hippocampal slow-oscillatory phase-triggered averages of LFPs from temporal lobe regions, showing rhythmic modulation of gamma-band activity. (G) Gamma amplitude averaged across hippocampal slow-oscillatory phase bins. (H) Phase-amplitude coupling differences for MTL gamma envelopes relative to hippocampal 1-, 2-, and 6-Hz rhythms, using 1-Hz as the reference. (I) Difference in phase-amplitude coupling contributions (MTL minus non-MTL regression coefficients). Bars indicate model coefficients ± 95% CIs. (J) Estimation plot showing differences in slow-oscillatory phase synchronization between MTL and non-MTL gamma activity patterns across task stages. (K) Phase-amplitude coupling differences for MTL gamma envelopes relative to hippocampal 2-Hz rhythm during (in-burst) versus outside (out-of-burst) 2-Hz burst events. (L) Estimation plot showing higher oscillatory phase synchronization in MTL versus non-MTL gamma activity patterns inside but not outside 2-Hz bursts. (M) Median differences in MTL neuron coactivity for in-burst versus out-of-burst periods at 1-, 2-, and 6-Hz (whiskers extend to 95% CIs). Statistics were assessed using two-sided paired permutation tests or linear mixed models where appropriate. Estimation plots (J and L) are as in Figure 1K. ***p < 0.001 and **p < 0.01; n.s., not significant.

We next asked whether this hippocampal architecture extends beyond the hippocampus to coordinate activity across the MTL network. To address this, we examined intracranial recordings from electrodes targeting other MTL regions (for example, the entorhinal cortex) as well as regions outside the MTL (for example, temporal cortices) (Figures 3A and 3B). As in the hippocampus (Figures 2F and S2M), single-neuron spike trains recorded across MTL regions exhibited slow-oscillatory rhythmicity centered near 2 Hz whereas neurons recorded outside the MTL displayed rhythmicity at other frequencies (Figure 3C). Spiking activity in MTL neurons was more strongly phase-locked to hippocampal slow-oscillatory phase (2-Hz) than to slower or faster rhythms (Figures 3D and 3E; 2-Hz versus 1-Hz, *p* = 0.002; 2-Hz versus 6-Hz, *p* < 0.001; 1-Hz versus 6-Hz, *p* = 0.059; two-sided paired permutation tests).

At the MTL network level, slow oscillatory power was most prominent in the hippocampus, whereas higher-frequency rhythmic activity dominated in extra-MTL regions (Figures S3A–S3C). The amplitude of 2-Hz oscillatory bursts did not correlate with ERP deflection in extra-hippocampal MTL regions (Figure S3D), a relationship that appeared specific to the hippocampus (Figure S1Q). Importantly, the hippocampal slow-oscillatory phase was associated with synchronization of gamma-band activity across MTL regions. Gamma activity patterns in extra-hippocampal MTL regions were more strongly coupled to hippocampal slow-oscillatory phase (2-Hz) than to slower or faster rhythms (Figures 3F–3H; 2-Hz versus 1-Hz, *p* < 0.001; 2-Hz versus 6-Hz, *p* < 0.001; 1-Hz versus 6-Hz, *p* = 0.320; two-sided paired permutation tests). Cross-frequency phase-amplitude coupling between hippocampal slow oscillatory activity and gamma patterns was stronger in MTL regions than in regions outside the MTL (Figures 3G and 3I; 2-Hz versus 1-Hz, *p* < 0.001; 2-Hz versus 6-Hz, *p* < 0.001; Wald tests on linear mixed-effects models).

This cross-regional synchronization was selectively enhanced during learning and recall compared with viewing (Figures 3J, S3E, and S3F; viewing, *p* = 0.455; learning, *p* = 0.004; recalling, *p* < 0.001; two-sided paired permutation tests). We then tested whether this coordination was preferentially expressed in slow-oscillatory burst events by comparing neuronal and network activity inside versus outside hippocampal 2-Hz bursts during learning and recall (“in-burst” versus “out-of-burst” activity; Figure 1L). Phase-amplitude coupling between hippocampal 2-Hz and MTL gamma-band activity was stronger inside than outside bursts (Figure 3K). Gamma activity across MTL regions also showed stronger phase alignment to hippocampal 2-Hz oscillations inside bursts (Figure 3L). At the population level, MTL neuron spiking coactivity was elevated during in-burst compared with out-of-burst periods (Figure 3M). Together, these results indicate that the hippocampal slow-oscillatory architecture coordinates neural activity across the MTL, with coordination concentrated within transient burst events that synchronize distributed population dynamics during human memory processing.

### Cross-regional spiking coactivity motifs structured by learning-evoked 2-Hz oscillatory bursts reactivate in post-learning hippocampal ripples

**Figure 4:**
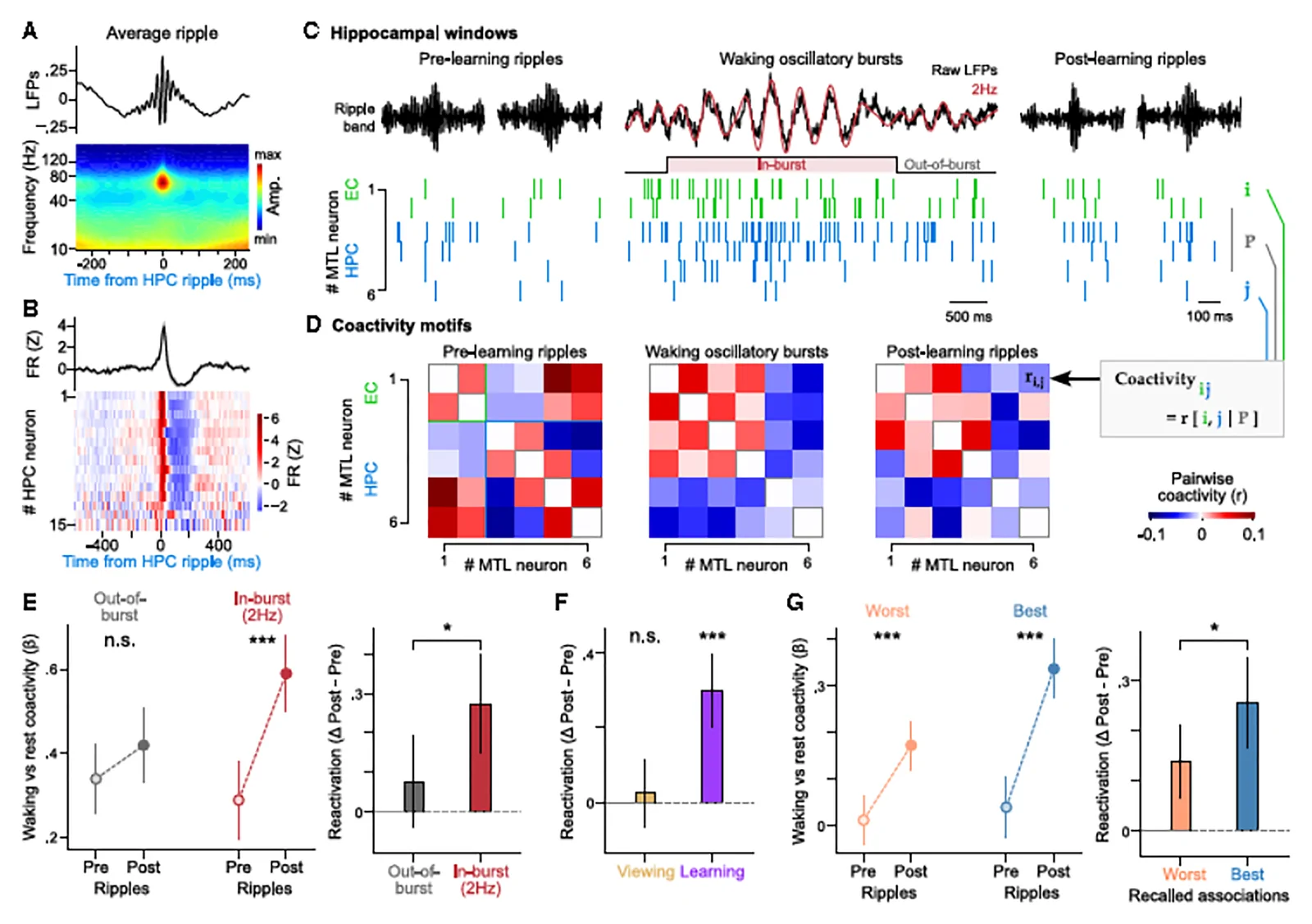
Cross-regional coactivity motifs organized by the hippocampal slow-oscillatory architecture are reactivated during offline ripples (A and B) Ripple-triggered average of hippocampal LFPs with corresponding spectrogram (A) and firing-rate heatmap of hippocampal neurons (B). Top traces in (A) and (B): mean ± SEM. (C and D) Reactivation of waking (viewing and learning) MTL coactivity motifs during hippocampal ripples. MTL population spike trains were extracted from hippocampal ripples during pre- and post-learning rest, as well as from waking 2-Hz bursts (in-burst) and periods outside these bursts (out-of-burst) during viewing and learning sessions (C). Coactivity motifs were derived from pairwise neuron-neuron (*i*,*j*) correlations after regressing out global population activity *P* (D). (E) Similarity between waking and ripple coactivity motifs quantified by generalized linear model (GLM) β coefficients (left), and corresponding reactivation strength (right). (F) Selective reactivation of learning-related but not viewing-related coactivity motifs. (G) Reactivation of coactivity motifs detected during learning trials associated with best versus worst subsequent memory recall performance. (E)–(G) show model coefficients ± 95% CIs. Statistical significance assessed using two-sided Wald tests on GLM coefficients; ***p < 0.001 and *p < 0.05; and n.s., not significant.

In animal models, population activity patterns formed during learning are later reactivated within hippocampal ripples to support memory consolidation during rest and sleep.^9,26,27,48–55^ Hippocampal ripples also occur in humans,^56–61^ where they could similarly support post-learning consolidation. To test whether an offline reactivation mechanism operates in humans, we examined hippocampal ripple activity during pre- and post-learning rest sessions (Figures 4A and S4A–S4C).

The human hippocampus exhibited ripples that occurred reliably during rest sessions, modulating neuronal spiking activity and displaying spectral and temporal properties comparable to those recorded during overnight sleep (Figures 4B and S4D–S4J). During slow-wave sleep, inter-ripple interval distribution showed a clear peak, reflecting consistent temporal spacing of ripple occurrence (Figures S4K–S4M). During rapid eye movement sleep, but not during slow-wave sleep, the human hippocampus also expressed 2-Hz activity similar to that observed during waking task engagement (Figures S4N–S4P). To assess post-learning reactivation, we extracted MTL population spiking patterns expressed during hippocampal slow-oscillatory bursts in viewing and learning sessions, and compared these waking in-burst patterns with population activity observed during hippocampal ripples in pre- and post-learning rest (Figure 4C). For comparison, we also extracted waking spiking patterns expressed outside slow-oscillatory bursts and compared these out-of-burst patterns with ripple activity across rest sessions.

For each task session, we computed MTL coactivity motifs using pairwise neuron-neuron correlations while regressing out the activity of the remaining population (Figure 4D). Offline reactivation was quantified using generalized linear models predicting ripple coactivity from waking motifs, with interaction terms capturing changes in waking-ripple similarity between post- and pre-learning rest.

Following learning, coactivity motifs structured by the slow oscillatory architecture were selectively reactivated during hippocampal ripples. Specifically, ripples exhibited significantly stronger reactivation for coactivity motifs expressed during slow-oscillatory bursts than for those expressed outside these bursts (Figure 4E; in-burst, *p* < 0.001; out-of-burst, *p* = 0.202; in-burst versus out-of-burst, *p* = 0.025; Wald tests) or for motifs associated with slower or faster rhythmic activity (Figure S4Q). Moreover, population coactivity evoked by photographic stimuli during learning reactivated more strongly during post-learning ripples than that evoked during viewing (Figures 4F and S4R–S4T; learning, *p* < 0.001; viewing, *p* = 0.622; learning versus viewing, *p* < 0.001; Wald tests). Critically, coactivity motifs associated with the best subsequent memory performance showed the strongest post-learning ripple reactivation (Figure 4G; best and worst recalled, both *p*s < 0.001; best versus worst recalled, *p* = 0.043; Wald tests).

Together, these findings demonstrate that population dynamics structured by the slow oscillatory architecture during learning are selectively reactivated during hippocampal ripples, linking online memory processing to offline consolidation in humans.

## Discussion

Our findings establish a unifying organizational principle for human memory in which slow-oscillatory burst events provide the temporal backbone that links online encoding, offline consolidation, and subsequent recall across the MTL. This architecture organizes population spiking, synchronizes gamma-band activity across hippocampal and extra-hippocampal regions, and structures patterns of neuronal coactivity that are selectively reactivated during offline hippocampal ripple events. Rather than reflecting a continuous background rhythm, it operates through transient, task-evoked oscillatory bouts that define windows for coordinated network interactions during memory processing.

Human hippocampal activity has long appeared variable,^42,43^ leaving unresolved whether oscillatory dynamics in the MTL provide an organizing framework for memory comparable to those described in animal models. Neuronal spiking analyses provide important support for probing oscillatory dynamics. Human intracranial recordings inherently permit simultaneous tracking of only a small number of units over time. Cluster-isolation criteria adapted from rodent-tetrode work, implemented here to ensure high confidence in unit identity and stability across task sessions, further reduce the number of retained single neurons. Intracranial recordings have nonetheless indicated slow-frequency oscillations in the human hippocampus, including activity in the ~2–4 Hz range associated with memory encoding and gamma coupling, whereas effects in the conventional rodent theta range are often weaker or variable.^39–41,47^ Other studies have linked theta-range power, entorhinal stimulation, thalamic stimulation, and cholinergic modulation to memory performance, highlighting the behavioral relevance of slow-frequency dynamics in human memory circuits.^44,62–64^ Our results provide a mechanistic framework that integrates these diverse observations by identifying a slow-oscillatory architecture that organizes MTL activity during memory processing in humans. In our data, 2-Hz burst events were selectively expressed during mnemonic engagement—that is, periods of active acquisition or retrieval of associations—rather than during passive viewing, distinguishing them from nonspecific fluctuations related to arousal or sensory processing.

In animal models, hippocampal oscillations provide a temporal framework for coordinating memory-related neural activity. In rodents, theta-band oscillations organize population spiking during active behavior, support interactions with nested gamma-band activity, and structure patterns of neuronal coactivity during learning.^15,19,20,22,23,25^ These patterns are subsequently reactivated during hippocampal ripples that occur during rest and sleep, supporting offline consolidation.^9,26,27,49–55^ Together, these findings have shaped influential models of memory and cognition in which oscillatory dynamics organize mnemonic processing, including frameworks that link online encoding and recall with offline consolidation.^7,10,31–34^

However, accumulating evidence indicates that hippocampal oscillatory dynamics vary substantially across species and behavioral contexts. In larger-brained mammals, including rabbits, cats, bats, and primates, hippocampal rhythmic activity is often slower, more intermittent, and more closely linked to task demands than to locomotion.^35–38^ In humans, reports of MTL oscillatory activity have emphasized its variability and intermittent nature,^42,43^ raising the question of whether such activity can provide an organizing architecture capable of coordinating neuronal spiking, network synchronization, and offline reactivation across memory states.

Our findings show that, despite their transient nature, 2-Hz bursts anchored in the hippocampus structure memory-related network dynamics across the human MTL. These bursts pace population spiking, synchronize gamma-band activity across hippocampal and extra-hippocampal regions, and organize co-activity motifs that are selectively reactivated offline during post-learning hippocampal ripples, with reactivation strength predicting subsequent recall accuracy. These coordinating effects occur within burst events that define discrete temporal windows for concentrated network interactions. They are expressed within a narrow frequency range centered at ~2 Hz, with weaker effects at adjacent slower (1-Hz) and faster (6-Hz) rhythms, indicating a constrained and functionally specific organizing time-scale rather than a generic property of broadband slow-frequency activity. Through this frequency-specific organization of network interactions, oscillatory coordination during learning is linked to offline consolidation and later memory performance.

This slow oscillatory architecture can be interpreted as a species-adapted organizing mechanism that preserves the logic of oscillatory coordination while operating at a slower timescale in the human brain. As brains enlarge across species, maintaining coherent timing among distributed circuits becomes increasingly challenging. Compensatory adaptations such as increased axon caliber and myelination help offset conduction delays introduced by longer conduction paths, preserving relative temporal relationships across regions.^65^ Within this context, a slower coordinating rhythm may facilitate communication across distributed networks without compromising faster nested dynamics for local computation. Consistent with this scaling framework, the peak frequency of the hippocampal theta rhythm, long implicated in waking memory processing in animal models, decreases with brain size across mammals, from ~5–12 Hz in mice and rats to ~3–7 Hz in bats, rabbits, cats, and primates.^10,17,35–38,66,67^ The identification of a slow organizing rhythm at ~2 Hz that paces online memory dynamics in humans extends this continuum without implying strict frequency homology. Moreover, hippocampal expression of this slow oscillatory architecture during rapid eye movement sleep (Figures S4N–S4P)^68,69^ further supports the idea that a conserved organizing logic operates across brain states as well as across species.

Our findings do not suggest that memory coordination in humans depends on a direct analog of rodent theta. Instead, they support a conserved organizing principle in which slow oscillatory dynamics structure population activity, coordinate crossregional communication, and link online encoding with offline consolidation. Together, these results show that human memory is organized by a scalable oscillatory architecture that coordinates network dynamics across learning, consolidation, and recall through mnemonic engagement-locked events. Without this transient, slow oscillatory architecture, a core principle of memory-related network organization in humans that links neuronal spiking, cross-regional communication, and ripple-mediated consolidation would remain mechanistically unexplained.

## Resource Availability

### Lead contact

Requests for further information, resources, and reagents should be directed to and will be fulfilled by the lead contact, David Dupret (david.dupret@bndu.ox.ac.uk).

### Materials availability

This study did not generate new, unique reagents.

### Data and code availability

- The electrophysiology dataset reported in this study is being used in ongoing projects and can be accessed under a data transfer agreement due to data protection requirements. We welcome inquiries for sharing it—please contact the lead contact.
- This paper does not report original code.
- Any additional information required to reanalyze the data reported in this paper is available from the lead contact upon request.

## Star★Methods

### Key Resources Table

**Table:** 

REAGENT or RESOURCE	SOURCE	IDENTIFIER
Software and Algorithms		
Pegasus Software (Toulouse)	Neuralynx	36-0301-0037 Version 2.2.2
Pegasus Software (Paris)	Neuralynx	36-0301-0034 Version 2.2.2
Cheetah Software (Paris)	Neuralynx	Version 1.1.0
FreeSurfer	Laboratories for Computational Neuroimaging, Athinoula A. Martinos Center	https://surfer.nmr.mgh.harvard.edu/
BrainVISA Morphologist	Fischer et al.^70^	https://brainvisa.info/web/morphologist.html
Kilosort via SpikeForest	Magland et al.^71^; Pachitariu et al.^72^	N/A
Empirical Mode Decomposition in Python	Quinn et al.^73^	https://doi.org/10.21105/joss.02977
Tailored Masked Empirical Mode Decomposition (tmEMD)	Clarke-Williams et al.^45^	https://doi.org/10.5281/zenodo.10351412
Mask frequencies for human LFP tmEMD	Adrien Causse et al., this study	https://doi.org/10.60964/rnd-14mc-v747
Other		
Atlas Neurophysiology System (Toulouse)	Neuralynx	16SX 256-channel
Atlas Neurophysiology System (Paris)	Neuralynx	16SX 160-channel
Dixi electrodes / Microdeep® Micro-macro (Toulouse)	Dixi Medical	MM08-09A33D08; MM08-09A40D08; MM08-09A51D08; MM08-06B33D12; MM08-06B40D12; MM08-06B51D12; MM08-06B40P12; MM08-06B33P12
Behnke-Fried electrodes / Macro electrodes (Paris)	Ad-Tech® Medical	SD04R-SP05X-000; SD06R-SP05X-000; SD08R-SP05X-000; SD10R-SP05X-000; SD12R-SP05X-000; BF09R-SP61X-0BB; WB09R-SP00X-014

### Experimental Model and Study Participant Details

#### Subjects

This study included a total of 35 adult participants [mean age (interquartile range): 35 (24–42) years; 16 males, 19 females; 26 right-handed] undergoing intracranial monitoring for pharmacologically intractable epilepsy. Participants were recruited from the Epilepsy and Sleep Unit of the Neurology Department at Toulouse University Hospital (Toulouse, France; n = 24) and from the Epilepsy and EEG Units at Pitié-Salpêtrière Hospital (Paris, France; n = 11). All patients underwent stereo-electroencephalography with multi-contact depth electrodes over 7–10 days to localize seizure foci. Electrode placement followed clinical requirements and pre-surgical trajectories determined from individual structural MRI scans. All participants provided informed consents following the procedures approved by the relevant institutional review boards.

### Method Details

#### Intracranial electrodes

Participants were implanted with standard and hybrid depth electrodes. In Toulouse, hybrid electrodes (DIXI Medical; maximum of six electrodes per subject; platinium/iridium, 2 mm in length, 0.8 mm in diameter) comprised 5–18 macrocontacts and were equipped with two or three tetrodes (tungsten wires, 20 μm in diameter), as described in Despouy et al.^46^ Tetrodes typically protruded between the two deepest macrocontacts on each electrode (Figure 1E); in few hybrid electrodes, they extended between the eighth and the ninth macrocontacts to sample more superficial regions. The reference for both tetrodes and macrocontacts was a macrocontact located in white matter. Bilateral implantations allowed recording from contralateral, unaffected sites (sentinel electrodes). In Paris, hybrid electrodes contained 9 macrocontacts (3 mm spacing between contact 1 and 2, and 4.5 mm for the others) and eight microwires (Behnke-Fried electrodes, Ad-Tech Medical; maximum of two electrodes per subject), with one additional microwire used as a reference. Standard electrodes had 5 mm spacing between contacts. The anatomical location of each macrocontact was determined by co-registering postoperative CT scans with preoperative 3D T_1_-weighted MRI data, as presented in Fernandez Vidal et al.^74^ Preoperative T1 3T MRI images were automatically segmented in the native space (using FreeSurfer, https://surfer.nmr.mgh.harvard.edu/), and the anatomical location of each electrode macrocontact was then detected on the CT scans and positioned in the Desikan-Killiani atlas.^70,75^ The location of all gray matter contacts and Behnke-Fried microwire bundles were further verified by an expert clinician. Each tetrode was assigned the anatomical label of the nearest macrocontact. To localize macrocontacts within the hippocampal volume, each macrocontact was assigned a standardized anatomical position based on individual hippocampal geometry. Hippocampal volumes were segmented from native T_1_-weighted MRI scans, and segmentation quality was manually verified (used in Figures S1A, S1J, S2A, and S4A). All contact coordinates were then normalized in the Montreal Neurological Institute (MNI) space for group-level analyses and visualizations (Figures 1F, 1G, 3A, and 3B). Hippocampal macrocontacts with y-axis coordinates < -22 in the MNI space were considered posterior (Figure S2E). Of 170 contacts, 105 were located in the right hemisphere.

#### Neurophysiological recordings

Electrophysiological signals were recorded continuously for approximately one hour while participants performed the behavioral task, using an ATLAS 16SX 256-channel Neuralynx (Toulouse) and ATLAS 16SX 160-channel Neuralynx (Paris). For overnight sleep, additional surface electrodes were also used according to the 10–20 system alongside electrooculography and electromyography electrodes for polysomnography.^76^ Data were filtered between 0.1 and 8,000 Hz and sampled at 32,768 Hz, then downsampled to 20,000 Hz and 1,250 Hz for subsequent analyses of population spiking and neural oscillations, respectively. Macrocontact and microelectrode signals were visually inspected to exclude artifactual channels. In total, 2,667 macrocontacts were retained (99 ± 24 per subject). A common-average referencing was applied to macrocontact data (using the median to minimize the potential influence of large-amplitude events including interictal epileptiform discharges) but not to microelectrode recordings. Where mentioned in the manuscript, bipolar referencing was occasionally used for control analyses (Figures S1H and S1I). Line noise at 50 Hz was minimal under the recording conditions; consequently, notch filtering was never applied. Synchronization between stimulus events (e.g., photographic presentations) and neural recordings was achieved via TTL pulses, with each event type assigned a unique TTL identification code. Throughout the manuscript, local field potentials (LFPs) refer to intracranial EEG signals recorded from macrocontacts, except in Figures 2A, S4B, and S4C where tetrode LFPs were used. LFPs from microwires (Behnke-Fried electrodes) are not used in this study because local referencing was employed for initial acquisition of the signal. Consequently, Behnke-Fried electrodes are not displayed in the figures of this manuscript.

#### Behavioral datasets

Twenty-seven participants (n = 16 in Toulouse; n = 11 in Paris) performed a computer-based associative memory task implemented in MATLAB using Psychophysics Toolbox 3 (PTB-3). The task involved learning associations among individuals forming a community, in which each person was represented by a photograph and a name. Each recording day consisted of five consecutive sessions: pre-learning rest, viewing, learning, post-learning rest, and recall (Figure 1A; total duration: ~1 hour). During the two rest sessions, participants sat still with eyes closed (10 minutes each session). In the viewing session (8 minutes), they were familiarized with 144 photographs of community members displayed for 1 second each and in random order, separated by 1.5–3 second inter-stimulus intervals sampled from a gamma distribution, during which a central fixation dot was shown. Participants were instructed to maintain fixation. To ensure compliance, we asked them to press the space bar of the computer keyboard whenever they detected that the same photograph was presented consecutively, a repetition that we introduced at random intervals. We then briefly familiarized participants to the structure of the learning session (1–2 minutes). During the learning session (10 minutes), participants viewed pairs of individuals presented consecutively, one person after the next, to associate them (i.e., “paired associates”). Between each pair, participants were instructed to maintain fixation on the displayed central dot (for 2 seconds as an inter-pair interval). Learning progress was assessed through intermittent multiple-choice questions: a cue photograph appeared for 1 second, followed by a screen displaying three possible associates. Participants had up to 10 seconds to identify the correct associate and respond using the left, down, or right arrow keys. In this community, one individual was intermittently shown holding a key to a treasure chest and participants were asked to remember it. After the post-learning rest session, participants performed the recall session (15 minutes) to test retention of the learned associations. During recall, participants answered two types of memory questions. The first type consisted of multiple-choice questions identical to those used during learning, in which participants identified the correct associate among three photographs. The second type presented a single photograph for 1 second, after which participants judged whether the depicted person could help obtain the key based on the associations they had learned. Responses were permitted after a 2-second delay, creating a fixed 3-second interval between cue onset and the earliest possible motor response. This delay ensured that the visual display was directly comparable to the viewing and learning photographs for analysis of event-related potentials and evoked oscillations (Figures 1J, 1K, and S1P–S1R). The recording day finished with a post-recall viewing session, which we used to confirm that the changes in oscillation power during the memory recall session were not merely reflecting passage of time (data not shown).

We further validated the network physiology identified in the awake human hippocampus during the relational memory task by examining data from eight additional awake participants who were watching a sitcom series (*Friends*), and were instructed to maintain attention throughout each episode (~25 minutes per episode). A member of the team continuously monitored participants’ vigilance to ensure constant attention.

To further validate our ripple detection in the human hippocampus during rest sessions of the relational memory task, we also characterized oscillatory patterns expressed during overnight sleep in seven of our participants recruited to do the memory task. These participants were recorded continuously for 24 hours including overnight sleep. Sleep staging was performed according to American Academy of Sleep Medicine guidelines.^76^ N2 and N3 stages were merged and referred to as slow-wave sleep throughout the manuscript.

#### Spike detection and unit isolation

Spike sorting and unit isolation were performed with an automated clustering pipeline using Kilosort (https://github.com/cortex-lab/KiloSort) via the SpikeForest and SpikeInterface frameworks (https://github.com/flatironinstitute/spikeforest),^71,72^ as previously used in rodent-tetrode studies.^21,25^ For this, the algorithm restricted templates to channels within a given microelectrode while masking all other recording channels. All sessions recorded contiguously on a given day were concatenated and cluster cut together to monitor cells throughout the experiment. The resulting clusters were verified by the operator using cross-channel spike waveforms, auto-correlation histograms, and cross-correlation histograms. Each unit used for analyses showed throughout the entire recording day stable spike waveforms, clear refractory period in their auto-correlation histogram, and absence of refractory period in its cross-correlation histograms with the other units. All analyses were performed on these single units. In total, 257 single units were identified in the MTL, of which 211 in the hippocampus (Figure S2L). All hippocampal single-units analyzed in this manuscript were detected using tetrodes unless stated otherwise (from n = 18 human participants out of the 24 recruited in Toulouse). This decision was made to ensure that hippocampal neurons were located close to the macrocontacts used for LFPs extraction (e.g., to detect the phase of local oscillations; see model of the hybrid electrode in Figure 1E). In the extra-hippocampal MTL, 39 single units were detected with tetrodes, and 7 using microwires (Behnke-Fried electrodes).

#### Behavioral analysis

Behavioral performance during learning and memory recall was quantified as the percentage of correct responses to multiple-choice questions. This was assessed intermittently across trials during the learning session (Figure 1B) and as the mean accuracy across all questions during the recall session (Figure 1C, Wilcoxon signed-rank test). To identify the best and worst recalled associations (Figures 1K, 1Q, and 4G), accuracy was calculated for each individual association within the learnt community. For each association, the mean accuracy across memory questions was computed. The two associations with the highest mean accuracy were labeled as “best”, and the two with the lowest mean accuracy as “worst”. Recording days when subjects exhibited ceiling performance across all associations were excluded from this analysis (n = 2 out of 34 recording days excluded). Participants who remembered correctly at least three quarters of the associations were defined as “higher performers” while the others were considered as “lower performers” (Figures S1Y and S1Z).

#### Decomposition of LFPs into oscillatory components

To decompose the local field potentials (LFPs) into their elementary oscillatory components, or intrinsic mode functions (IMFs), we applied tailored masked Empirical Mode Decomposition (tmEMD, https://doi.org/10.5281/zenodo.10351412; https://github.com/cjcw/tmEMD).^45,73^ EMD is an unsupervised, iterative sifting algorithm that extracts signal components based on local time-frequency properties. Masked EMD (mEMD) enhances this process by introducing a mask signal at each sifting step to reduce mode mixing and thus improve the separation of IMFs. The tailored variant of mEMD was developed to minimize mode mixing, increasing consistency across recordings from different subjects and electrode contacts. Using the macrocontacts located in the anatomical region of interest (e.g., hippocampus in Figures S1D–S1F), we applied the tmEMD procedure to LFP recordings using two steps. The first step allowed assessing the epochs to be analyzed and optimizing the masks. For each signal, we used 2 × 45-s LFPs epochs free of interictal epileptiform discharges (IEDs) and with high signal-to-noise ratio (SNR). To quantify SNR for a given epoch, we computed the Welch PSD and calculated the power ratio between 1–20 Hz and >200 Hz. Mask frequencies were optimized by pooling the identified clean epochs across macrocontacts and applying tmEMD to identify mask parameters that maximize consistency and minimize mode mixing (mask frequencies for human LFP tmEMD; https://doi.org/10.60964/rnd-14mc-v747). The second step performed the full-session decomposition using optimized masks: once optimal masks were identified, we applied mEMD to the full recording session. Instantaneous amplitude, frequency, and phase were then obtained using the Hilbert transform. IMF consistency was assessed by computing the PSDs of each IMF in IED-free epochs (Figures S1D and S1F). To characterize the frequency band of each IMF, we computed its PSD using Welch’s method and averaged the resulting spectra across all macrocontacts (Figure S1E thick lines). For each IMF, the peak frequency was defined as the frequency of maximum power, and the frequency band was determined as the contiguous band containing 80% of the total spectral power (i.e., 80% power band).

#### Power spectral densities

Power spectral density (PSD) estimates were computed using Welch’s method with a Hann window (from the scipy.signal.welch function in the scipy.signal module). PSDs were calculated across contiguous 1-s windows with a frequency-dependent sampling density optimized for slow and fast oscillations (20 points/Hz for 0.5–20 Hz; 4 points/Hz for 20–200 Hz). All LFPs signals were z-scored prior to spectral estimation (Figures 1H, S4B, and S4N). To account for interindividual differences in the broadband (aperiodic) component of the power spectrum, we modeled each PSD using the “Fitting Oscillations and One-Over-F” (FOOOF) algorithm.^77^ Each PSD was fit within the 0.5–20 Hz range to isolate the aperiodic (1/f) component. The full modeled spectrum and the aperiodic fit were then subtracted to obtain a log-corrected, aperiodic-adjusted PSD, preserving only periodic features (Figures S1I, S1K–S1N, S3A–S3C, and S4O). Narrowband oscillations were then quantified by averaging power within the 1-Hz (0.5–1.25 Hz), 2-Hz (1.25–3.5 Hz), and 6-Hz (3.75–8.5 Hz) frequency bands, corresponding to the power distribution of the initially detected IMFs (Figure S1E). In addition, to characterize rhythmicity in single-neuron spiking activity, PSDs were computed on spike-train autocor-relograms. For each unit, the autocorrelogram was calculated over a 2-s window and smoothed with a Gaussian kernel (width = 10 ms, σ = 5 ms) to reduce high-frequency noise while preserving rhythmic structure. The PSD of the resulting autocorrelation signal was then computed (0.9–15 Hz range) to identify oscillatory modulation of firing patterns. Peaks in these autocorrelogram-derived PSDs indicate dominant rhythmic frequencies in the spike timing of individual neurons (Figures 2F, 3C, and S2M).

#### Detection of interictal epileptiform discharges (IEDs)

We detected IEDs recorded in our datasets using an envelope-based, data-driven algorithm adapted from Janca et al.^78^ The continuous LFPs traces were downsampled to 200 Hz and band-pass filtered (Chebyshev type-II high-pass 10 Hz and low-pass 60 Hz, stopband attenuation 30 dB). The analytic envelope (Hilbert) was computed and scanned with overlapping 5-s windows stepped by 1 second. Within each window, the envelope distribution was modeled by a log-normal function, and events were detected when the signal amplitude exceeded three times the typical (mode + median) level estimated from that distribution. This adaptive thresholding identifies transient amplitude outliers that deviate from the typical background activity. Window thresholds were cubic-spline interpolated to sample resolution and boxcar-smoothed, and contiguous supra-threshold samples were grouped into events. Nearby events were merged by extending boundaries by ±120 ms and the envelope peak within each merged window was taken as the IED time. All detections were produced on the downsampled signal and indices were mapped back to the native sampling rate for downstream analyses. Examples of detected events are shown in Figure S1J. Macrocontacts showing an average detection rate below one IED per minute during active sessions were considered free of epileptiform activity.

#### Comparison of oscillatory power between task sessions

Session-related changes in oscillatory power were quantified across task sessions for the 1-, 2-, and 6-Hz frequency bands. Power values were obtained from log-corrected PSDs computed for each hippocampal macrocontact as described above (see section “power spectral densities“), excluding those with interictal activity (Figures S1L–S1N). Linear mixed-effects models were used to compare power across sessions, with task session treated as a fixed effect and individual subject as a random factor, allowing assessment of frequency-specific modulation of slow oscillations throughout the task, controlling for putative subject-driven effects (Figure S1N).

#### Comparison of event-related potentials between sessions

Event-related potentials (ERPs) were measured by averaging LFPs time-locked to photograph onsets. For each recording, signals were z-scored, and trials contaminated by IEDs or falling outside the recordings were excluded. Epochs extending from 1 second before to 4 seconds after photograph onset were extracted, baseline-corrected using the mean pre-stimulus activity, and averaged across trials for each contact. To test for session-related differences, the two average ERPs were compared using a nonparametric cluster-based permutation test (1,024 iterations, mne-python package^79^), which identifies contiguous time intervals showing consistent ERP deflections differences while controlling for multiple comparisons. Significant clusters (p < 0.01) indicated time windows with reliable modulation of evoked responses across sessions (Figure S1O).

#### Wavelet spectrograms and quantification of evoked oscillations

Amplitude spectrograms were computed from the raw signal using complex Morlet wavelet convolution for each macrocontact across sessions. This approach provides high temporal precision for fast frequencies and high frequency resolution for slow oscillations, making it well suited to characterize non-stationary neural dynamics. Wavelets were constructed with five cycles per frequency and normalized to unit energy, producing 80 logarithmically spaced frequencies between 1 and 150 Hz. Stimulus-locked spectrograms were then extracted from –1 to +3 seconds (viewing and recalling sessions) or +4 seconds (learning session) relative to stimulus onset, excluding trials containing IEDs or motor responses. To ensure comparability across sessions, trials were sub-sampled to equalize stimulus counts. For each contact and frequency, post-stimulus amplitudes were normalized to baseline (–1 to 0 second) by z-scoring and averaged across trials using the amplitude from the baseline only (Figures 1J, S1Q, S1R, and S3D). This baseline normalization was not applied in Figure S1P, where the mean amplitudes in the post-stimulus window were compared to mean amplitudes obtained from the baseline. Mean evoked amplitudes were then quantified within the 1-, 2-, and 6-Hz frequency bands during the post-stimulus window (+1 to +3 seconds for viewing and recalling, +2 to +4 seconds for learning). This ensures that the observed changes in evoked amplitude are not confounded by differences in the magnitude of ERPs deflection between conditions.

#### Correlation between ERP deflection and evoked oscillatory amplitude

To relate ERPs deflection with evoked oscillations in the recalling session, we compared the mean ERP deflection (0.3–0.9 second after stimulus onset in the hippocampus) with the corresponding post-stimulus spectral amplitude averaged within the 1-, 2-, or 6-Hz bands (1–4 second window). Correlations were computed across contacts using Pearson’s coefficient, and significance was assessed by two-tailed testing. Scatterplots in Figures S1Q and S3D display the linear fit with bootstrapped 95% confidence intervals, showing the relationship between the strength of evoked slow oscillations and the magnitude of the ERP deflection. The mean ERP deflection was taken between 0.3–0.9, 0.15–0.6 and 0.15–0.5 second after stimulus onset for the entorhinal cortex, parahippocampal cortex and amygdala macrocontacts, respectively (Figure S3D). These epochs were selected to match the biggest ERP deflection visible in the grand averages computed for each region. However, various epochs were tested and none of them showed significant correlation with evoked amplitude (*data not shown*).

#### Detection and quantification of oscillatory bursts

Oscillatory bursts (transient bouts) were detected from IMF-derived signals using a spectrogram-based thresholding approach. For each hippocampal recording, the signal reconstructed up to the IMF of interest (1-, 2- or 6-Hz IMF) was converted to a wavelet spectrogram using 30 logarithmically spaced (0.2–20 Hz for 1-Hz bursts, 0.5–25 Hz for 2-Hz bursts, and 1–45 Hz for 6-Hz bursts). The spectrogram was z-scored, and contiguous time-frequency clusters exceeding a z-score of 2 were identified as candidate bursts. Clusters were retained only if at least half of their energy fell within the target frequency band (0.5–1.5, 1–4, 5–12 Hz for 1-, 2- and 6-Hz bursts, respectively) and if their duration corresponded to at least two oscillatory cycles (or four for measures of burst rate). Adjacent clusters separated by <400 ms (1-Hz), <200 ms (2-Hz) or <50 ms (6-Hz) were merged. Example 2-Hz bursts are shown in Figures 1L and 4C. Burst onset and offset were then defined from the merged clusters, and each burst’s mean duration (cycle count) was extracted (Figures 1M and S1S). Out-of-burst epochs corresponded to all segments between detected bursts (see Figures 1L and 4C). Burst rate of occurrence and burst duration were compared between viewing, learning and recall sessions using paired statistics (Figures 1N, 1O, S1U, and S1W); between best- and worst-recalled associations using paired statistics (Figure 1Q); and between higher and lower performers using unpaired statistics (Figures S1Y and S1Z). To characterize the temporal dynamics of burst rate (Figures 1P and S1X) and duration (Figures S1T and S1V) across the task, each session for each participant was divided into 50 equal-duration time bins. Burst rate and duration per bin were then calculated by averaging across hippocampal contacts and participants. This “warped time” was used to compute the Pearson correlation between burst rate or duration and task progression within each session. To estimate burst dynamics across the task (Figures 1P, S1T, S1V, and S1X), the mean bin duration for each session was used to rescale the bins. Accordingly, although each session is represented by 50 time bins, the relative temporal proportions of each session are preserved.

#### Detection of gamma activity

To detect gamma activity, the signal was divided into five contiguous frequency bands between 60 and 160 Hz (20-Hz steps). Each band was bandpass-filtered and transformed into its analytic representation using the Hilbert transform to obtain the instantaneous amplitude. The envelope from each band was normalized by its mean amplitude to equalize their contributions, and the normalized envelopes were averaged across all bands to produce a single broadband gamma envelope representing moment-to-moment fluctuations in gamma activity (Figure 2A).

#### Detection of individual oscillatory cycles

To segment individual oscillatory cycles from each intrinsic mode function (IMF), we identified four key waveform features: troughs (local minima), peaks (local maxima), ascending and descending zero-crossings. Cycles were defined as sequences of six temporally ordered points: two consecutive troughs (or alternatively, two peaks, or two zero-crossings), and four intermediate landmarks corresponding to characteristic inflection points within the waveform (e.g., zero-crossings and extrema). Each cycle was retained only if all six points followed a strictly increasing temporal order, ensuring well-formed and physiologically plausible waveforms. Additional checks were applied to exclude cycles with overlapping or missing components. This procedure enabled the consistent identification of complete cycles across IMFs (Figures 2B, 3F, S2A, and S2C).

#### Oscillatory cycle-triggered averages

To relate slow oscillatory phase (1-, 2-, and 6-Hz) to gamma activity and population firing rate (Figures 2B and S2C), we computed cycle-triggered averages aligned to each extracted IMF cycle. Cycles that coincided with IEDs were excluded. For each cycle, we extracted ±2.5 seconds around the descending zero-crossing and binned signals at 10, 5, or 3 ms resolution for 1-, 2-, and 6-Hz cycles, respectively. LFPs were z-scored, and spike counts were converted to population rate (Hz) per bin. Cycle-averaged LFP, gamma, spike-rate traces, and time–frequency spectrograms (>10 Hz) were obtained by averaging across cycles (and across units for the population rate). To visually compare the three frequencies, gamma activity was normalized, and the same y-scales were applied to all graphs (Figures 2B and S2C).

#### Phase–amplitude coupling and spike–field locking

To quantify the actual phase–amplitude coupling (PAC) between gamma activity and slower oscillations, we computed the modulation index (MI) as in Tort et al.^80^ Gamma signals were taken either from the same macrocontact signal as the phase (local PAC; Figures 2E, S2E, and S2F) or from a distal macrocontact (distal PAC, across structures; Figures 3H and 3I). The computation of the MI involved measuring the deviation of the amplitude distribution across phase bins from uniformity using the Kullback–Leibler divergence. First, we measured the instantaneous phase of the IMFs using the Hilbert transform. The phase of each IMF (e.g., 2-Hz) was then binned into *N* equally spaced intervals over [0, 2π]. For each bin *i*, we computed the mean amplitude of the gamma activity *A[n]* as:

$$\bar{A_{i}}=\frac{1}{\left|S_{i}\right|}\underset{n\in S_{i}}{\sum }A[n],$$

where *S*_*i*_ is the set of time points for which *ϕ[n]* falls into bin *i*. We then normalized the amplitude distribution:

$$P(i)=\frac{\bar{A_{i}}}{\sum_{j=1}^{N}\bar{A_{j}}}.$$

The Kullback–Leibler divergence between this distribution and the uniform distribution $U(i)=\frac{1}{N}$ was computed as:

$$D_{\text{KL}}(P\|U)=\sum_{i=1}^{N}P(i)\log\left(\frac{P(i)}{U(i)}\right).$$

We normalized this value to define the modulation index:

$$\text{MI}=\frac{D_{\text{KL}}(P\|U)}{\log(N)}.$$

This yielded a value between 0 (no coupling; uniform distribution of amplitudes across phase bins) and 1 (maximal concentration in a single phase bin), indicating how strongly the amplitude of the gamma activity depends on the phase of the slow oscillation. Then, we generated 300 surrogate versions of the intrinsic mode functions (IMFs) while preserving their spectral content by applying phase randomization in the Fourier domain. This approach destroys temporal structure but retains the power spectrum, making it suitable for surrogate-based statistical testing of the PAC.

The original signal *x[n]* was transformed using the discrete Fourier transform (DFT):

$$X[k]=\sum_{n=0}^{N-1}x[n]e^{-2\pi ikn/N},$$

where *A[k]* is the amplitude spectrum and *ϕ[k]* the phase spectrum. This was expressed in polar form as:

$$X[k]=A[k]e^{i\phi [k]},$$

where *A*[*k*] = |*X*[*k*]| is the amplitude spectrum, and *ϕ*[*k*] = arg(*X*[*k*]) is the phase spectrum.

We then generated a random permutation *π(k)* over frequency indices and substituted:

$$\tilde{X}[k]=A[k]e^{i\phi [\pi (k)]}.$$

The inverse Fourier transform yielded the phase-randomized surrogate signal:

$$\tilde{X}[n]=\text{R}\left\{\frac{1}{N}\sum_{k=0}^{N-1}\tilde{X}[k]e^{2\pi ikn/N}\right\}.$$

We then computed phase–amplitude coupling (PAC) between the unchanged gamma activity and each of the 300 surrogate phase signals. This generated a null distribution of PAC values, which we used to assess statistical significance. The actual PAC value, computed using the original (non-randomized) phase signal, was transformed into a z-score relative to this surrogate distribution. This z-scored PAC reflects the likelihood—rather than the magnitude—of coupling and was used for statistical comparisons across IMFs and electrode contacts (Figures 2E, 3H, and S2E).

To assess the relationship between spike timing and the phase of slower oscillations, we computed the pairwise phase consistency (PPC) as in Vinck et al.^81^ Spike trains were either taken from neurons located in the same region as the phase (local PPC, nearest macrocontact; Figures 2C and 2D) or from a distal region (distal PPC, across structures; Figures 3D and 3E). For each neuron, we extracted the instantaneous phase of the IMF (e.g., 2-Hz) at the time of spike occurrence. Given a set of *N* spike times, each associated with a phase value *ϕ*_*n*_ ∈ [0, 2*π*] the PPC is computed as:

$${\text{PPC}}_{0}=\frac{2}{N(N-1)}\sum_{i=1}^{N}\sum_{j=i+1}^{N}\cos\left(\phi_{i}-\phi_{j}\right).$$

This expression corresponds to the average cosine of the phase differences between all unique spike pairs. It can also be reformulated for computational efficiency using trigonometric identities:

$${\text{PPC}}_{0}=\frac{2}{N(N-1)}\sum_{i=1}^{N}\sum_{j=i+1}^{N}\left[\cos\left(\phi_{i}\right)\cos\left(\phi_{j}\right)+\sin\left(\phi_{i}\right)\sin\left(\phi_{j}\right)\right].$$

The PPC value ranges from −1/(N−1) to 1; it is centered around 0 for uniformly distributed phases; 1 indicates perfect phase locking; and can take negative values due to finite-sample variability or phase configurations dominated by large pairwise phase differences. To determine whether the observed spike–phase coupling was statistically significant, we used the same phase randomization followed by z-scoring procedure described above for PAC. Specifically, we generated 300 surrogate phase signals by shuffling the phase time series and recomputed the PPC between each surrogate phase and the spike times. This yielded a null distribution, against which the PPC computed from the original phase signal was z-scored. This z-scored PPC reflects the likelihood of spike–phase locking beyond chance and was used for statistical testing across IMFs and neuronal units (Figures 2C, 2D, 3D, and 3E). A z-score threshold of 5 was used to identify significant coupling. Preference was then measured by comparing, for each given neuron, which of the three oscillations (1-, 2-, or 6-Hz) had the highest z-scored PPC value, only considering neurons with at least one significant locking in these frequency bands (Figure 3E, right). Both PAC and PPC were measured on epochs clear from interictal discharges.

#### Quantification of phase reversal

Laminar phase relationships along depth electrode contacts were analyzed to identify polarity reversals between adjacent hippocampal recording sites. Average LFPs were first computed from three neighboring macrocontacts, aligned to the positive peaks of 2-Hz cycles detected on the deepest contact (e.g., “Hpc 1a” in Figure S2A). The 2-Hz instantaneous phase was then extracted from IMFs on each macrocontact using the Hilbert transform, and circular statistics were applied to determine the mean phase difference between all contact pairs (Figure S2B).

#### Cross-correlation between gamma activity and population firing rate

To assess the temporal coupling between local neuronal firing and gamma activity, we computed time-lagged cross-correlations between population rate and gamma activity. For each recording session, we retained hybrid electrodes located in the hippocampus and with at least five recorded neurons. Population rate was obtained by summing spike trains from all single-units recorded within the same region and temporally smoothing the resulting spike-rate vector with a Gaussian kernel (width = 50 ms, σ = 25 ms). For each region, Pearson correlation coefficients were computed between the population rate and each local gamma trace across contiguous, IEDs-free epochs. To evaluate specificity, the same population rate signal was correlated with gamma envelopes from distal macro-contacts located on other electrode shafts. Mean correlation coefficients across distal contacts were used as controls (Figure S2D).

#### Anatomical gradients in local phase–amplitude coupling

Anatomical gradients in local phase–amplitude coupling (PAC) were assessed to determine whether the strength of oscillatory coupling varied along hippocampal axes. For each hippocampal contact, coupling values were related to the anteroposterior and mediolateral coordinates of this recording site using a generalized linear model fitted separately for the 1-, 2-, and 6-Hz frequency bands. Coupling values were transformed to match normality of the distributions (Yeo-Johnson):

$${\text{PAC}}_{i}=\beta_{0}+\beta_{1}A{\text{P}}_{i}+\beta_{2}{\text{ML}}_{i}+\varepsilon_{i},$$

where PAC_*i*_ is the transformed local phase–amplitude coupling value for contact *i*; AP_*i*_ and ML_*i*_ are the anteroposterior and medio-lateral coordinates of this recording site; *β*_0_ is the intercept; *β*_1_and *β*_2_ represent the effects of position along the hippocampal axes; and *ε*_*i*_ is the residual error term. Model coefficients were extracted and summarized as a heatmap (Figure S2F).

#### Amplitude modulation using Holo-Hilbert Spectral Analysis (HHSA)

The HHSA is designed to analyze non-linear and non-stationary signals and capture both carrier frequencies and their amplitude modulations.^82^ This spectral method uses a two-layer EMD followed by Hilbert transforms to provide a two-dimensional representation of energy across carrier frequencies and modulation frequencies. This allows dealing with non-linearities and harmonics and reduces the risk of detecting spurious PAC^83^ to identify genuine cross-frequency interactions and nested oscillations in LFPs signals. To do this, we first selected epochs clear from interictal epileptic discharges and used IMFs extracted using tmEMD on each macro-contact, retaining the first *K=9* IMFs capturing slow to fast oscillations. Each IMF was transformed into its analytic signal via the Hilbert transform:

$$Z_{k}(t)={\text{IMF}}_{k}(t)+i\cdot ℋ\left[{\text{IMF}}_{k}(t)\right],$$

yielding instantaneous amplitude *A*_*k*_(*t*) = |*z*_*k*_(*t*)| and carrier frequency:

$$f_{k}^{\left(1\right)}(t)=\frac{1}{2\pi }\frac{d}{dt}\arg\left(z_{k}(t)\right).$$

To capture amplitude fluctuations over time, we applied a second-layer EMD to the amplitude envelopes, decomposing each into slower amplitude modulation components:

$$A_{k}(t)=\sum_{j=1}^{J}a_{k,j}(t).$$

Each modulation component was again Hilbert-transformed to extract modulation frequency.

The holospectrum represents signal energy jointly as a function of carrier frequency *f*^(1)^ and amplitude modulation frequency f ^(2)^. It was computed across all IMF pairs and projected onto a 2D frequency space:

$$H\left(f_{\text{carrier}},f_{\text{AM}}\right)=\text{Energy}\;\text{at}\left(f^{\left(1\right)},f^{\left(2\right)}\right).$$

Log-spaced frequency bins were used from 0.1 to 195 Hz for both axes, allowing fine resolution of slow-frequency modulations. The average energy measured on each electrode contact signals were then z-scored before averaging across contacts. We finally averaged the energy in several frequency bands to compare gamma (60–160 Hz) modulation across slow oscillations (Figures S2G–S2K) That is, in humans, 1-Hz (0.5–1.25 Hz), 2-Hz (1.25–3.5Hz), and 6-Hz (3.75–8.5 Hz); in mice, 3-Hz (1.5–4.5 Hz), 7-Hz (5–10 Hz), and 15-Hz (11–20 Hz). To quantify the gain in amplitude modulation in contacts clear from interictal discharges, we computed the paired difference between 2-Hz and 6-Hz energy per macrocontact (Figure S2I).

#### Identification of putative pyramidal neurons and interneurons

Putative pyramidal neurons (pPYR) and interneurons (pINT) were distinguished based on spike waveform features extracted from individual hippocampal units. To improve resolution, the 32 waveform points sampled at 20 kHz were upsampled by a factor of 100 using quadratic interpolation (via the interpolate function from scipy). For each neuron, trough-to-peak latency and peak amplitude asymmetry were computed from the average normalized spike waveform. Trough-to-peak latency was defined as the time interval between the negative trough and the subsequent positive peak of the waveform. Peak amplitude asymmetry was quantified as (*b* − *a*)/(*b* + *a*), where *a* and *b* correspond to the amplitudes of the pre- and post-trough positive peaks (also named “shoulders” of the spike waveform), respectively (Figure S2N, left). Clustering was performed using a Gaussian mixture model (two components) applied to the joint distribution of trough-to-peak latency and peak amplitude asymmetry. Clusters were interpreted as putative pyramidal neurons (pPYR; broad spikes) and putative interneurons (pINT; narrow spikes).^25^ Example phase preference at 2 Hz between pPYR and pINT recorded from the same tetrode are represented in Figure S2O. For each tetrode containing both pPYR and pINT units, pairwise phase consistency at 2 Hz was averaged within each class (pPYR or pINT), and these values were compared between classes using paired statistics (Figure S2P).

#### Comparison of oscillatory power between brain regions

Regional differences in oscillatory power were assessed by comparing log-corrected spectra between hippocampal (HPC), extra-hippocampal medial temporal (MTL; entorhinal, parahippocampal, amygdala), and non-MTL temporal cortical contacts (inferior, middle and superior temporal cortices and fusiform gyrus). For each macrocontact, log-corrected spectra were computed as described in theSTAR Methods section *“*power spectral densities*“* (Figure S3A). To better capture the dominance of alpha oscillations over 2-Hz in non-MTL regions, we then used the peak power measured as the maximum of the corrected power spectrum in the designated frequency band (Figures S3B and S3C). To quantify the relative predominance of 2-Hz over 6-Hz activity, a normalized ratio (2Hz - 6Hz) / (2Hz + 6Hz) was computed for each macrocontact and compared across regions (Figure S3C).

#### Mixed-effects models for regional differences in distal phase–amplitude coupling

To test whether distal phase–amplitude coupling to HPC differed between MTL and non-MTL contacts and whether that difference depended on frequency, we pooled distal PAC estimates for the 1-, 2- and 6-Hz bands across macrocontacts and subjects and fitted a mixed-effects model with an MTL×frequency interaction and recording day as a random factor. Coupling values were transformed for normality (Yeo-Johnson) and distance between contacts was included as a covariate:

$${\text{PAC}}_{ij}=\beta_{0}+\beta_{1}{\;\text{MTL}}_{i}+\beta_{2}\;\text{Frequency}+\beta_{3}\left({\text{MTL}}_{i}\times \text{Frequency}\right)+\beta_{4}\;{\text{Distance}}_{ij}+u_{ij}+\varepsilon_{ij},$$

where PAC_*ij*_ is the transformed distal phase–amplitude coupling value for the pair of distal macrocontact *i* and hippocampal contact *j*; MTL_*i*_ is a binary variable indicating whether contact *i* is in the medial temporal (MTL) or not; *Frequency* represents the categorical frequency factor (1-, 2-, or 6-Hz); Distance_*ij*_ is the Euclidean distance between contact *i* and *j* and controls for inter-contact spacing; *u*_*ij*_ is the random intercept accounting for subject-level variability; and *ε*_*ij*_ is the residual error term. From the fitted model we extracted the MTL [non-MTL simple effect at each frequency (estimated difference ± 95% CI) and tested difference-of-differences contrasts (2-Hz versus 1-Hz and 6-Hz) using Wald *t*-tests. This method quantifies whether MTL contacts show selectively greater slow-frequency coupling and whether that regional bias is specific to the 2-Hz band (Figure 3I).

#### Estimation of phase-gamma pattern synchronization

Phase synchronization between gamma activity recorded from different macrocontacts was quantified from the dispersion of their preferred hippocampal 1-, 2-, and 6-Hz phases. For each hippocampal macrocontact, the mean preferred phase of gamma activity was determined for all extra-hippocampal sites, and the pairwise circular differences between these phases were computed. The average of these pairwise differences was then inverted (2π – Δ phase) so that higher values reflected stronger phase alignment. Synchronization indices were calculated separately for MTL and non-MTL contacts to compare the spatial organization of phase coupling across regions (Figures 3J, 3L, S3E, and S3F).

#### Detection of hippocampal ripples

Hippocampal ripple events were identified and validated through a multistage procedure combining automated detection, dimensional embedding, and template-based classification (from section “*Initial ripple detection using overnight sleep recordings*“ to section “*Template matching and white-matter control*“).

#### Initial ripple detection using overnight sleep recordings

Hippocampal LFPs recorded during slow-wave sleep were bandpass-filtered between 60 and 180 Hz, and ripple candidates were identified by thresholding the ripple-band amplitude and comparing it to the closest white-matter macrocontact (referred to as control). Candidate events were retained when the amplitude in the ripple band exceeded five standard deviations and was at least twice as strong than in the control macrocontact, and the event lasted for at least one oscillatory cycle. The precise peak times of the filtered signal were extracted to provide a first set of putative ripples for each macrocontact during overnight sleep.

#### Dimensional embedding and template generation

For each recoding night, candidate ripple waveforms were then extracted from the ripple-band–filtered signal (60–180 Hz) using 25-ms snippets centered on the ripple peaks. These band-limited segments, capturing the oscillatory component of each event, were normalized and used as input to Isomap (15 neighbors, intrinsic dimensionality estimated per recording night) to embed the high-dimensional waveforms into a low-dimensional space while preserving their temporal structure.^84^ Events were clustered with k-means, and clusters corresponding to canonical ripple morphologies were manually verified and retained. Then, within each recording night, 200 waveforms from verified clusters were subsampled and averaged to form “super-events”. These “super-events” were pooled across recording nights and clustered again to generate a compact library of representative ripple templates characterized by their average waveform and dominant frequency. Dominant frequency of the templates was estimated from the inverse of the period between two oscillatory peaks in the average ripple-band–filtered signal. Fourteen different templates were retained, ranging from dominant frequency 67.6 Hz to 113.6 Hz.

#### Loose detection of putative ripple events

A permissive detector was then applied to all hippocampal recordings to capture all potential high-frequency bursts in the ripple range (60–180 Hz). The analytic amplitude envelope was smoothed (width = 80 ms, σ = 40 ms) and adaptively thresholded to detect transient events, targeting 20–30 events per minute while constraining event duration (≥20 ms) and separation (≥150 ms). Periods containing IEDs were excluded to prevent contamination by large, transient high-power deflections and to avoid detecting pathological events. Event peaks were realigned using the instantaneous phase of an 80-Hz narrowband signal to ensure consistent phase definition.

#### Template matching and white-matter control

These loosely detected events were then compared to each validated ripple template using cosine similarity. For every event, the highest similarity score and corresponding template were obtained both in the hippocampal contact and in its anatomically defined white-matter control by realigning to the local peak in the ripple-band. Events were classified as genuine ripples only when their match score exceeded 0.85 in the hippocampal macrocontact but remained below this threshold in the control macrocontact. This match score of 0.85 corresponded to the 95^th^ percentile of the distribution of all match scores obtained in white matter macro-contacts, representing a meaningful null distribution for transient, non-specific fast-frequency events. This two-stage validation—requiring both high morphological similarity to verified sleep ripples and spatial specificity relative to the white-matter reference—ensured that the final set of detected events reflected true, locally generated hippocampal ripples.

#### Ripple-triggered averages

Ripple-triggered averages were computed to characterize hippocampal LFP, spike train, and spectral dynamics as well as associated gamma activity from the MTL contacts during rest and sleep sessions. For each hippocampal contact, detected events were realigned to the local peak of the ripple-band signal to ensure consistent phase alignment across ripples. Around each realigned peak, ±250 ms of broadband LFP and corresponding time–frequency spectrograms (10–200 Hz, logarithmic spacing) were extracted and averaged across events to obtain mean ripple-locked waveforms (Figures 4A, S4C, and S4G). In Figure 4B, spike trains of hippocampal neurons recorded using microwires (Behnke-Fried electrodes) during overnight slow-wave sleep in 3 participants were averaged in 12-ms bins, ±600 ms around ripples, z-scored per neuron (bottom panel) and averaged across neurons (top panel).

In Figure S4C, ripples were detected on the macrocontact and realigned to the local peak of the ripple-band signals obtained from the local and the distal tetrodes. In Figure S4G, ripple-triggered averages were computed on the same example hippocampal macro-contact, from events detected in the task rest or during overnight N1 sleep.

#### Characterization of ripple central frequency

Hippocampal ripple events were analyzed from LFPs band-pass filtered between 55 and 200 Hz (zero-phase fourth-order Butter-worth filter). Instantaneous phase and amplitude were obtained using the Hilbert transform. Ripple onset and offset were defined as the first and last time points at which the z-scored amplitude exceeded 2. Within these limits, the unwrapped phase was used to compute the total number of oscillatory cycles as the difference between phase values at offset and onset (in degrees) divided by 360. For instance, an unwrapped phase change of 1800° corresponds to five cycles (1800/360 = 5). The ripple central frequency was then calculated by dividing the number of cycles by the event duration in seconds (e.g., 3 cycles / 400 ms = 75 Hz). This analysis was performed separately in rest (Figures S4F and S4J) and sleep recordings (Figures S4F and S4H), with sleep events further grouped by sleep stage (wake, N1, SWS, REM).

#### Characterization of inter-ripple intervals

Inter-ripple intervals were measured by taking the delay between consecutive ripples detected using hippocampal macrocontacts (humans) or dCA1 tetrodes (mice). Representative examples of inter-ripple intervals are shown in Figure S4A (humans) and S4L (mice). Inter-ripple intervals were then aggregated across subjects and recording sessions (6 humans recorded overnight sessions, 12 mice recorded in one-hour sleep sessions) and represented as histograms in Figures S4K and S4M. Kernel density estimates were obtained from these pooled distributions (Figures S4K and S4M, black curves) and from individual recording sessions (Figures S4K and S4M, grey curves).

#### Comparison of oscillatory power between sleep stages

State-dependent changes in slow oscillatory power were quantified across sleep stages (Figure S4O; SWS, N1, REM, and wake) for the 1-, 2-, and 6-Hz frequency bands. Continuous LFPs recordings were divided into 30-s epochs based on manual sleep scoring, and log-corrected PSDs were computed as described above (see section *“*power spectral densities*“*). To ensure comparable sampling across stages, 200 randomly selected windows were analyzed per condition. For wakefulness, only daytime segments (8:00 a.m. to 6:00 p.m.) were included, excluding brief night-time arousals.

#### Detection of cross-regional neuronal coactivity motifs

Pairwise coactivity motifs were derived from simultaneously recorded single-unit activity during in-burst versus out-of-burst periods (Figures 3M, 4E, and S4Q), task epochs (Figures 4F and S4R–S4T) and hippocampal ripples. Bursts were detected as described above (see section “*Detection and quantification of oscillatory bursts*“) for 1-, 2- and 6-Hz IMFs. For each recording day, units from the MTL were included when at least five well-isolated neurons were available. Spike trains were converted into binned firing-rate matrices using 25-ms bins. We then selected bins overlapping with oscillatory bursts (Figure 4E, viewing and learning sessions concatenated), photograph presentation and its subsequent inter-stimulus intervals in task (viewing versus learning, best versus worst conditions, Figures 4F and 4G) or ripple events (±200 ms). Control bins were drawn from out-of-burst epochs (Figure 4E) or out-of-ripple intervals of matched duration and distance from ripple events (*data not shown*). Firing rates were z-scored for each neuron, and the influence of overall population activity was removed by regressing out the instantaneous population rate (*P*). Pairwise coactivity between neurons *i* and *j* was defined as the Pearson correlation between their residual binned firing rate (Figure 3M), producing one coactivity matrix per condition. To assess the specificity of these patterns, neuron identities were randomly permuted within each time bin, preserving the instantaneous population profile but disrupting pairwise structure. Coactivity matrices computed from the permuted data provided null distributions (Figure S4T). For confirmation, we verified that hippocampal ripples propagated in MTL structures during SWS and N1 sleep (*data not shown*).

#### Reactivation of neuronal coactivity motifs in hippocampal ripples

Reactivation was quantified by relating neuronal coactivity motifs expressed during wake (evoked by photographs or expressed during oscillatory bursts) to those measured during pre-learning and post-learning ripples. Pre-learning ripples constitute a necessary control to isolate experience-dependent reactivation from intrinsic ripple-related synchrony.^9,20,26,27,30,48,85^ Without this control, an increase in neuronal coactivity could be attributed to offline reactivation of the wake experience (e.g., learning) while in fact it may instead reflect the intrinsically high synchrony of hippocampal ripples,^55^ which can occur in the absence of experience-specific reactivation.^86^ For each recording day, the neuron-by-neuron coactivity matrices were vectorized and concatenated across participants to form predictor and response variables (n = 603 coactivity motifs from 6 participants with at least 5 simultaneously recorded MTL neurons). Group-level relationships were estimated using generalized linear models (GLMs).

For each waking condition, the following model (Model 1):

$$Y=\beta_{0}+\beta_{\text{wake}}X_{\text{wake}}+\beta_{\text{cond}}C_{\text{rest}}+\varepsilon$$

was fitted to quantify the correspondence between waking and rest motifs, where *Y* is the vector of ripple coactivity coefficients, *X*_wake_ the corresponding waking coefficients (in-burst or out-of-burst, viewing or learning, best or worst recalled associations), and *C*_rest_a categorical factor indicating pre- or post-learning rest. Parameter estimates, 95% confidence intervals, and p-values were obtained from Wald t-tests on fitted GLM coefficients. Model 1 provided β coefficients (*β*_cond_) and their 95% confidence intervals reported in Figures 4E, 4G (left panels), and S4Q (left panel) and are referred to as “Waking vs rest coactivity (β)”.

Reactivation strength was then defined as the change in the slope linking waking and ripple motifs across rest sessions, estimated from the interaction term in the following model (Model 2):

$$Y=\beta_{0}+\beta_{\text{wake}}X_{\text{wake}}+\beta_{\text{cond}}C_{\text{rest}}+\beta_{\text{int}}\left(X_{\text{wake}}\times C_{\text{rest}}\right)+\varepsilon .$$

The interaction coefficients (*β*_int_) and their 95% confidence intervals extracted from Model 2 represent the difference between post- and pre-learning wake–rest slopes and are referred to as “Reactivation (Δ Post – Pre)” in Figures 4E, 4G (right panels), and S4Q (right panel). The *p*-value of these interaction coefficients (*β*_int_) was used to assess significance of the reactivation in Figures 4E–4G (left panels) and S4Q (left panel).

Differences in reactivation between waking conditions (in-burst vs out-of-burst, viewing vs learning, best vs worst recalled associations) were tested with a combined model (Model 3) including a three-way interaction term *X*_wake_ × *C*_rest_ × *C*_cond_, where *C*_cond_ indexes the waking condition. The *p*-value of this three-way interaction coefficient was used to assess significance in Figures 4E and 4G (right panels) and S4Q (right panel).

At the subject level (Figure S4R), reactivation was assessed as the partial correlation between waking and ripple coactivity vectors computed separately for each recording day, controlling for the alternate rest session (prelearning controlling for post-learning rest and vice versa).

### Quantification and Statistical Analysis

Data analyses were conducted using Python version 3.10, incorporating the following packages: DABEST v2023.2.14,^87^ scikit-learn v1.2.2 and nilearn v0.10.1,^88^ NumPy v1.24.3,^89^ SciPy v1.10.1,^90^ Stats-Models v0.14.0,^91^ Matplotlib v3.7.1,^92^ Pandas v1.5.3,^93^ and Seaborn v0.11.0,^94^ MNE-Python v1.5.1.^79^ Symmetric distribution assumptions underpinned the two-sided statistical tests, visualized using Gardner-Altman and Cumming plots from the DABEST Framework (e.g. Figures 1K, 3J, and S2D). These plots illustrate effect sizes by comparing mean or median differences across groups. Each plot consists of two panels: the top (or left) shows raw data distributions with group means ± SEM (unless stated otherwise), and the bottom (or right) shows differences relative to a reference group, calculated from 5,000 bootstrapped samples. Black dots represent the mean (or median), black ticks indicate 95% confidence intervals, and bootstrapped error distribution curves are included. To compare two conditions, bootstrap tests were employed. These tests, which accommodated both paired and unpaired comparisons, estimated the bootstrapped mean difference (either absolute or as a percentage relative to one of the two variables) by resampling the data 100,000 times (unless stated otherwise) with replacement. For paired comparisons, indices were resampled to preserve the relationship between pairs, whereas for unpaired comparisons, each condition was resampled independently. P values for these tests were computed numerically, under the null hypothesis of zero difference. The p value was determined by multiplying the smaller proportion of bootstraps below or above zero by two. Median differences were preferred over mean differences when the original distributions were skewed (visual inspection). In some instances (e.g. Figures 1J, 2D, 2E, 3E, 3H, 3K, and 3M), we visualized the bootstrapped mean (or median) differences using histograms or boxplots, from which the corresponding p values were derived. These histograms do not depict the distribution of raw data points but rather represent empirical estimations of the sampling distribution of the mean difference, obtained by the resampling approach described above. All confidence intervals (95% CI) were calculated via bootstrapping with 100,000 resamples (unless stated otherwise). For each interval, data were resampled randomly with replacement, and the 2.5th and 97.5th percentiles of the bootstrapped distributions determined the lower and upper bounds of the CI. Two-sided *t*-tests or Wilcoxon signed-rank tests were also used to compare conditions, depending on whether normality (assessed by the Shapiro–Wilk test) was met. Significance of model coefficients was evaluated using two-sided Wald tests.

## Supplementary Material

**Supplemental Information**

Supplemental information can be found online at https://doi.org/10.1016/j.neuron.2026.05.004.

Supplemental Figures and Tables

## In brief

Causse et al. identify a learning-evoked slow-oscillatory architecture in the human medial temporal lobe. Transient hippocampal 2-Hz bursts pace neuronal spiking and synchronize gamma-band activity across the MTL, structuring population coactivity motifs during learning. These coactivity motifs are selectively reactivated during hippocampal ripples during post-learning rest, and reactivation strength predicts subsequent recall, linking encoding, consolidation, and retrieval through a unified coordination mechanism.

## Highlights

- Learning elicits transient 2-Hz slow-oscillatory bursts in the human hippocampus
- These bursts pace neuronal spiking and synchronize gamma activity across the MTL
- Burst-structured coactivity motifs are reactivated in post-learning ripples
- Post-learning reactivation strength predicts subsequent memory recall
