# Supplementary material for: A learning-evoked slow-oscillatory architecture paces population activity for offline reactivation across the human medial temporal lobe
Source: Neuron. Author manuscript; Available in PMC 2026 Jun 13. (PMC7619154; doi:10.1016/j.neuron.2026.05.004)
Supplement: Supplemental Figures and Tables [file EMS213816-supplement-Supplemental_Figures_and_Tables.pdf]

A learning-evoked slow-oscillatory architecture paces population activity for offline reactivation across the human medial temporal lobe

Adrien A. Causse[1,2,*], Jonathan Curot[3,4], Vítor Lopes-dos-Santos[1,2], Raphaël Nunes-da-Silva[1], Helen C. Barron[1,2,5], Vincent Dornier[3], Marie Denuelle[3,4], Amaury De Barros[6,7], Jean-Christophe Sol[6,7], Jean-Albert Lotterie[6,7], Katia Lehongre[8], Sara Fernandez-Vidal[8], Valerio Frazzini[9,10], Vincent Navarro[9,10], Luc Valton[3,4], Emmanuel J. Barbeau[3], Tim Denison[1,2], Leila Reddy[3,*], and David Dupret[1,2,11,*]

[1]Brain Network Dynamics Unit, Nuffield Department of Clinical Neurosciences, University of Oxford; Oxford, United Kingdom.
[2]Medical Research Council Centre of Research Excellence in Restorative Neural Dynamics; United Kingdom.
[3]CerCo, CNRS UMR5549, University Toulouse; Toulouse, France.
[4]Brain Electrophysiology, Epilepsy and Sleep Unit, Neurology Department, Toulouse University Hospital; Toulouse, France.
[5]Oxford Centre for Integrative Neuroimaging, University of Oxford, FMRIB, John Radcliffe Hospital; Oxford, United Kingdom.
[6]Department of Neurology and Neurosurgery, Toulouse University Hospital; Toulouse, France.
[7]Toulouse Neuro Imaging Center, INSERM, U1214; Toulouse, France.
[8]Centre de Neuro-Imagerie de Recherche, ICM Paris Brain Institute, Pitié-Salpêtrière Hospital; Paris, France.
[9]Sorbonne Université, Paris Brain Institute, ICM, Inserm, CNRS, Pitié-Salpêtrière Hospital; Paris, France.
[10]Assistance Publique-Hôpitaux de Paris, Epilepsy and EEG Units and Reference Center of Rare epilepsies, ERN EpiCare, Pitié-Salpêtrière Hospital; Paris, France.
[11]Lead Contact

*Correspondence: adrien.causse@ndcn.ox.ac.uk, leila.reddy@cnrs.fr, and david.dupret@bndu.ox.ac.uk.

Supplementary Figures

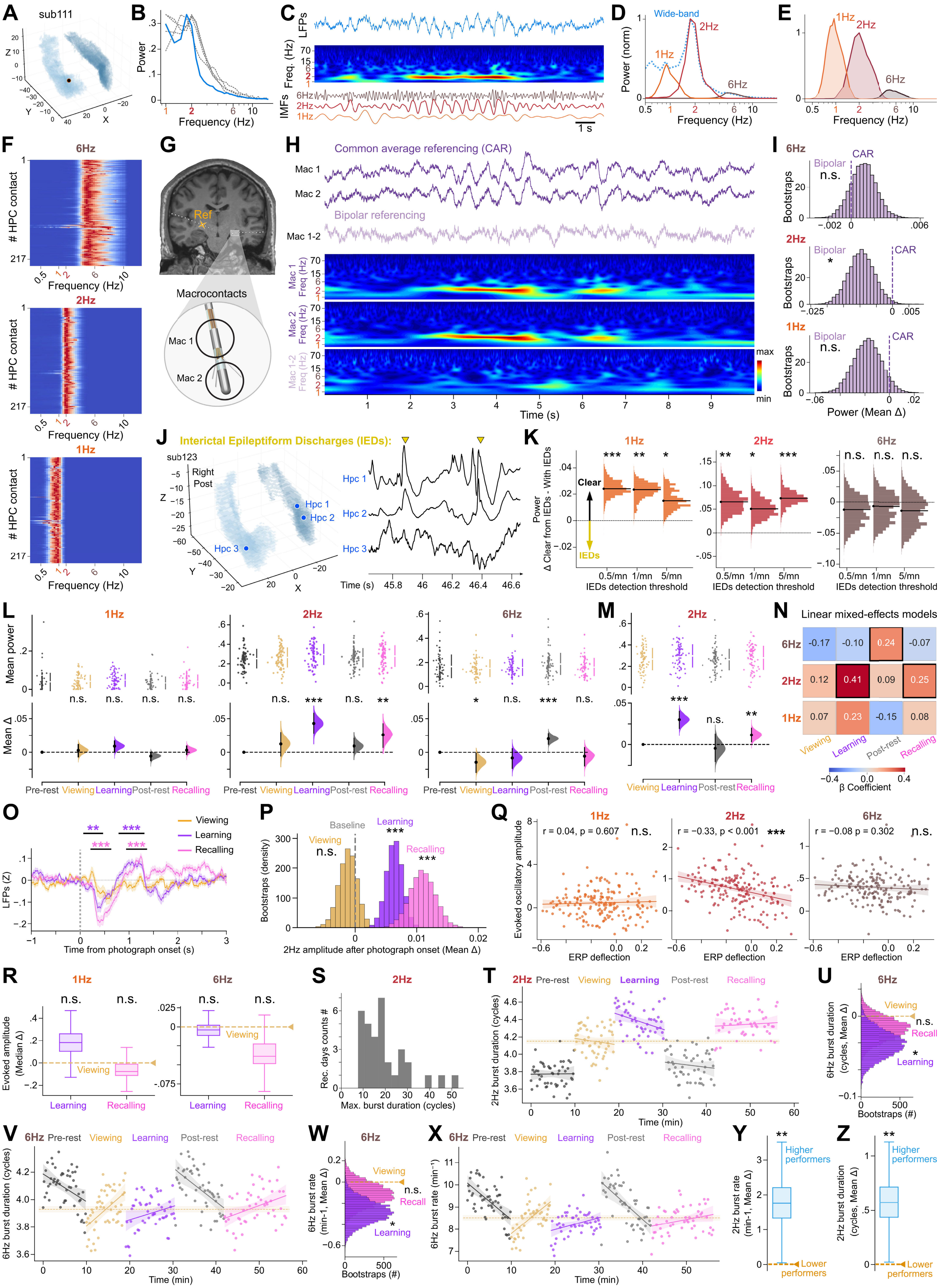
A
sub111
Z
Y
X
B
Power
Frequency (Hz)
C
LFPs
Freq. (Hz)
IMFs
6Hz
2Hz
1Hz
1 s
D
Wide-band
2Hz
1Hz
6Hz
Power (norm)
Frequency (Hz)
E
1Hz
2Hz
6Hz
Frequency (Hz)
F
6Hz
HPC contact
Frequency (Hz)
2Hz
HPC contact
Frequency (Hz)
1Hz
HPC contact
Frequency (Hz)
G
Ref
Macrocontacts
Mac 1
Mac 2
H
Common average referencing (CAR)
Mac 1
Mac 2
Bipolar referencing
Mac 1-2
Mac 1 Freq (Hz)
Mac 2 Freq (Hz)
Mac 1-2 Freq (Hz)
max
min
Time (s)
I
6Hz
Bipolar
CAR
n.s.
Bootstraps
2Hz
Bipolar
CAR
*
Bootstraps
1Hz
Bipolar
CAR
n.s.
Bootstraps
Power (Mean Δ)
J
Interictal Epileptiform Discharges (IEDs):
sub123
Right
Post
Hpc 1
Hpc 2
Hpc 3
Z
Y
X
Hpc 1
Hpc 2
Hpc 3
Time (s)
K
1Hz
***
**
*
Clear
IEDs
Power
Δ Clear from IEDs - With IEDs
0.5/mn
1/mn
5/mn
IEDs detection threshold
2Hz
**
*
***
0.5/mn
1/mn
5/mn
IEDs detection threshold
6Hz
n.s.
n.s.
n.s.
0.5/mn
1/mn
5/mn
IEDs detection threshold
L
1Hz
Mean power
n.s.
n.s.
n.s.
n.s.
Mean Δ
Pre-rest
Viewing
Learning
Post-rest
Recalling
2Hz
n.s.
***
n.s.
**
Pre-rest
Viewing
Learning
Post-rest
Recalling
6Hz
*
n.s.
***
n.s.
Pre-rest
Viewing
Learning
Post-rest
Recalling
M
2Hz
***
n.s.
**
Viewing
Learning
Post-rest
Recalling
N
Linear mixed-effects models
6Hz
-0.17
-0.10
0.24
-0.07
2Hz
0.12
0.41
0.09
0.25
1Hz
0.07
0.23
-0.15
0.08
Viewing
Learning
Post-rest
Recalling
-0.4
0.0
0.4
β Coefficient
O
**
***
***
***
Viewing
Learning
Recalling
LFPs (Z)
Time from photograph onset (s)
P
Baseline
Learning
***
Viewing
n.s.
Recalling
***
Bootstraps (density)
2Hz amplitude after photograph onset (Mean Δ)
Q
1Hz
r = 0.04, p = 0.607
n.s.
Evoked oscillatory amplitude
ERP deflection
2Hz
r = −0.33, p < 0.001
***
ERP deflection
6Hz
r = −0.08 p = 0.302
n.s.
ERP deflection
R
1Hz
n.s.
n.s.
Evoked amplitude (Median Δ)
Viewing
Learning
Recalling
6Hz
n.s.
n.s.
Viewing
Learning
Recalling
S
2Hz
Rec. days counts #
Max. burst duration (cycles)
T
2Hz
Pre-rest
Viewing
Learning
Post-rest
Recalling
2Hz burst duration (cycles)
Time (min)
U
6Hz
Viewing
n.s.
Recall
*
Learning
6Hz burst duration (cycles, Mean Δ)
Bootstraps (#)
V
6Hz
Pre-rest
Viewing
Learning
Post-rest
Recalling
6Hz burst duration (cycles)
Time (min)
W
6Hz
Viewing
n.s.
Recall
*
Learning
6Hz burst rate (min-1, Mean Δ)
Bootstraps (#)
X
6Hz
Pre-rest
Viewing
Learning
Post-rest
Recalling
6Hz burst rate (min⁻¹)
Time (min)
Y
**
Higher performers
Lower performers
2Hz burst rate (min-1, Mean Δ)
Z
**
Higher performers
Lower performers
2Hz burst duration (cycles, Mean Δ)

Figure S1. Characterization of slow oscillations in human hippocampus. *Related to Figure 1*

(A-C) The human hippocampus exhibits 2-Hz oscillations during waking behavior. **(A)** Example 3D projection showing electrode contacts in the hippocampus of one participant. **(B)** Power spectral densities (PSDs) from hippocampal recordings in waking task sessions reveal a prominent oscillation peak around 2-Hz in individual participants (*blue curve*, participant shown in A and C; *gray curves*, other example participants). All contacts included were free of interictal epileptiform discharges (IEDs). **(C)** Example recording showing 2-Hz oscillations (top), corresponding spectrogram (middle), and constituent oscillatory components identified by tailored masked empirical mode decomposition[1] (intrinsic mode functions, IMFs; see D-F).

(D-F) Tailored masked empirical mode decomposition of hippocampal LFPs. **(D)** Example PSD of the wide-band signal (dashed line) and corresponding 1-, 2-, and 6-Hz IMFs from a single electrode contact. **(E)** Average PSD across all hippocampal contacts, normalized to maximal 2-Hz power (thick lines: 80% power bands). **(F)** Heatmaps showing power distributions of the 1-Hz (peak [80% power band (PB)]: 1.08 (0.65–1.50) Hz), 2-Hz [peak (80% PB): 2.38 (1.25–3.50) Hz], and 6-Hz [peak (80% PB): 6.15 (3.75–8.50) Hz] IMFs across all hippocampal contacts.

(G-I) Local referencing reduces detection of slow oscillations. **(G)** T1-weighted MRI showing hippocampal contacts (top) and schematic of local referencing (bottom). Monopolar signals were acquired using a distal white-matter contact and re-referenced either using a common average reference (CAR; median across contacts) or by bipolar subtraction of adjacent contacts (e.g., Mac 1 minus Mac 2). **(H)** Example recording showing LFP traces referenced using CAR (purple) or local bipolar referencing (pink), with corresponding spectrograms. **(I)** Mean difference in hippocampal 1-, 2-, and 6-Hz power after bipolar referencing relative to CAR.

(J, K) Slow oscillation amplitude decreases at hippocampal contacts with higher interictal discharge rates. **(J)** 3D hippocampal volume showing three recording sites used in **K** (left) and example simultaneous recordings from these contacts (right). Yellow arrowheads: IEDs detected on the Hpc 1 contact. **(K)** Median differences in 1-, 2-, and 6-Hz power between contacts free of IEDs and those with IEDs, plotted as a function of IED-rate threshold. Spearman correlation between IED rate and 2-Hz power: $r = -0.25$, $P < 0.001$; and 6-Hz power: $r = 0.06$, $P > 0.336$.

(L-N) Hippocampal 2-Hz power increases during learning and recall. **(L)** Estimation plot showing mean power differences for hippocampal 1-, 2-, and 6-Hz oscillations across task sessions relative to pre-learning rest. **(M)** Same format as **L**, but relative to viewing. **(N)** Heatmap of β coefficients from a linear mixed-effects model predicting hippocampal 1-, 2-, or 6-Hz power as a function of task session (pre-learning rest as reference), with subject modeled as a random effect; black squares indicate significant coefficients (Wald test, $p < 0.05$).

(O-R) Event-related modulation of hippocampal slow oscillations. **(O)** Group-level average LFPs aligned to photograph onset during viewing, learning, and recall; stronger ERPs are observed during learning and recall. **(P)** Mean differences in post-stimulus 2-Hz amplitude relative to pre-stimulus baseline across task sessions. **(Q)** Correlation between ERP deflection and evoked oscillatory amplitudes at 1-, 2-, and 6-Hz during recall. **(R)** Median differences in evoked 1-Hz (left) and 6-Hz (right) amplitude during learning or recall relative to viewing (computed over post-ERP epochs >1 s after photograph onset).

(S) Distribution of maximal burst duration across participants (mean burst duration [95% confidence interval (CI)]: 19.6 [15.8 – 23.4] cycles per burst).

(T) Time course showing expression dynamics of hippocampal 2-Hz burst duration in the relational memory task, averaged across participants and contacts. Burst duration was negatively

correlated with time as learning progressed (learning, r = -0.33, P = 0.020; other task sessions, r > -0.120, P > 0.399).

(U-X) Dynamics of hippocampal 6-Hz bursts in the relational memory task. **(U)** Mean difference in 6-Hz burst duration for learning and recall relative to viewing. **(V)** Time course showing expression dynamics of hippocampal 6-Hz burst duration averaged across participants and contacts. **(W)** Mean difference in 6-Hz burst rate for learning and recall relative to viewing. **(X)** Time course showing expression dynamics of hippocampal 6-Hz burst rate averaged across participants and contacts.

(Y, Z) Mean difference in 2-Hz burst rate (Y) and duration (Z) in the learning session between higher and lower task performers.

Data were analyzed using two-sided paired permutation tests except in **K** and **O** where unpaired tests and cluster-based permutation tests were applied, respectively; ***P < 0.001, **P < 0.01, *P < 0.05; n.s., not significant.

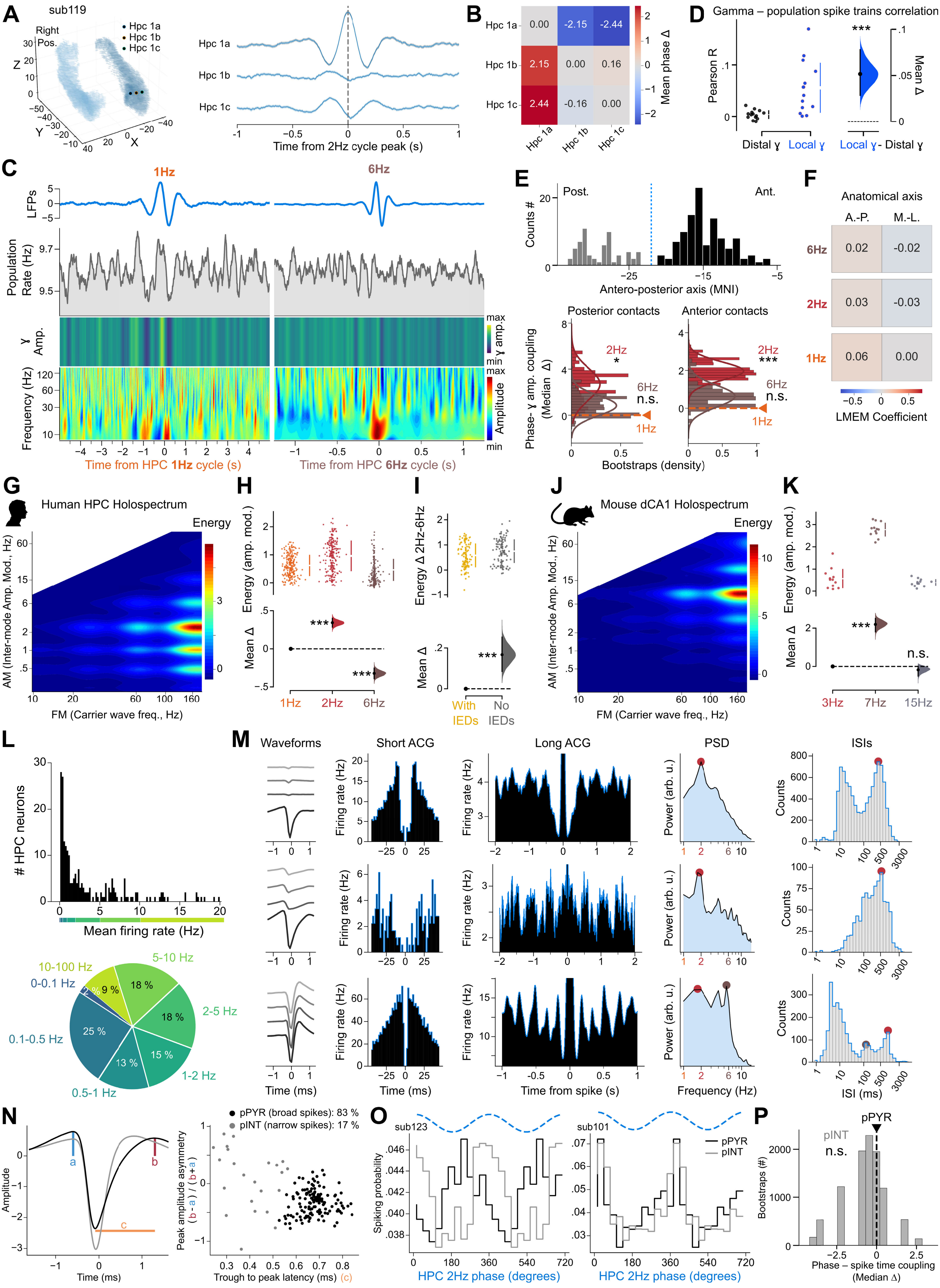
A
sub119
Right
Pos.
Hpc 1a
Hpc 1b
Hpc 1c
Time from 2Hz cycle peak (s)
B
Mean phase Δ
Hpc 1a
Hpc 1b
Hpc 1c
0.00
-2.15
-2.44
2.15
0.00
0.16
2.44
-0.16
0.00
D
Gamma – population spike trains correlation
Pearson R
Mean Δ
***
Distal ɣ
Local ɣ
Local ɣ - Distal ɣ
C
1Hz
6Hz
LFPs
Population Rate (Hz)
ɣ Amp.
max
min
ɣ amp.
Frequency (Hz)
Amplitude
Time from HPC 1Hz cycle (s)
Time from HPC 6Hz cycle (s)
E
Post.
Ant.
Counts #
Antero-posterior axis (MNI)
Posterior contacts
Anterior contacts
Phase- γ amp. coupling (Median Δ)
2Hz
*
6Hz
n.s.
1Hz
2Hz
***
6Hz
n.s.
1Hz
Bootstraps (density)
F
Anatomical axis
A.-P.
M.-L.
6Hz
0.02
-0.02
2Hz
0.03
-0.03
1Hz
0.06
0.00
LMEM Coefficient
G
Human HPC Holospectrum
Energy
AM (Inter-mode Amp. Mod., Hz)
FM (Carrier wave freq., Hz)
H
Energy (amp. mod.)
Mean Δ
***
***
1Hz
2Hz
6Hz
I
Energy Δ 2Hz-6Hz
Mean Δ
***
With IEDs
No IEDs
J
Mouse dCA1 Holospectrum
Energy
AM (Inter-mode Amp. Mod., Hz)
FM (Carrier wave freq., Hz)
K
Energy (amp. mod.)
Mean Δ
***
n.s.
3Hz
7Hz
15Hz
L
HPC neurons
Mean firing rate (Hz)
10-100 Hz
5-10 Hz
0-0.1 Hz
2 %
9 %
18 %
18 %
2-5 Hz
0.1-0.5 Hz
25 %
13 %
15 %
1-2 Hz
0.5-1 Hz
M
Waveforms
Short ACG
Long ACG
PSD
ISIs
Firing rate (Hz)
Power (arb. u.)
Counts
Time (ms)
Time from spike (s)
Frequency (Hz)
ISI (ms)
N
Amplitude
a
b
c
Time (ms)
pPYR (broad spikes): 83 %
pINT (narrow spikes): 17 %
Peak amplitude asymmetry (b - a) / (b + a)
Trough to peak latency (ms) (c)
O
sub123
sub101
Spiking probability
pPYR
pINT
HPC 2Hz phase (degrees)
HPC 2Hz phase (degrees)
P
pPYR
pINT
n.s.
Bootstraps (#)
Phase – spike time coupling (Median Δ)

Figure S2. Coupling of neuronal spiking and gamma-band activity to hippocampal slow oscillations. *Related to Figure 2*

(A, B) Phase reversal of hippocampal 2-Hz oscillations. **(A)** 3D hippocampal volumes showing three linearly arranged recording contacts from the same electrode shaft (left; Hpc 1a, 1b, and 1c) and corresponding average LFPs aligned to hippocampal 2-Hz oscillatory peaks (right), revealing polarity reversal across adjacent contacts. Shaded areas indicate mean ± SEM. **(B)** Heatmap of average phase differences between the three contacts shown in A. The observed reversal across linearly arranged contacts is reminiscent of cross-layer phase shifts described for hippocampal oscillations in animal models[2,3].

(C-F) Gamma activity coupling to hippocampal oscillations. **(C)** Average hippocampal LFPs aligned to 1-Hz (left) or 6-Hz (right) phases (blue trace) with corresponding instantaneous population rate, gamma amplitude, and spectrogram (see Figure 2B for comparison). **(D)** Estimation plot showing the difference in instantaneous correlations between neuronal spiking and gamma envelopes recorded at distal versus local macrocontacts. **(E)** Distribution of hippocampal contacts along the antero-posterior axis (top; anterior sites in black, posterior sites in gray) and median phase–amplitude coupling (z-score) differences between 1-Hz and 2- or 6-Hz oscillations across posterior (bottom left) and anterior (bottom right) contacts. Consistent 2-Hz preference is observed in both regions. **(F)** Heatmap of β coefficients from linear regression predicting phase–amplitude coupling (z-score) to 1-, 2-, or 6-Hz oscillations as a function of antero-posterior or medio-lateral contact position. No coefficients reached significance.

(G-K) Additional Holo-Hilbert spectral analysis further supports gamma amplitude modulation by 2-Hz oscillations in the human hippocampus. **(G)** Holospectrum averaged across time, IMFs, and hippocampal macrocontacts (z-scored), revealing preferential modulation of fast-frequency signals by 2-Hz oscillations in humans. **(H)** Estimation plot showing mean differences in amplitude modulation between 1-Hz and 2- or 6-Hz oscillations across hippocampal macrocontacts. **(I)** Estimation plot showing stronger 2-Hz versus 6-Hz modulation in contacts free of interictal discharges (IEDs) compared with those containing IEDs. **(J)** For comparison, holospectrum averaged over hippocampal CA1 contacts in mice[4], revealing preferential modulation of fast-frequency signals by ~7-Hz oscillations. **(K)** Quantification of **J** as in **H**, showing dominant amplitude modulation at ~7 Hz in mice (n = 12 sessions, 6 mice).

(L, M) Firing-rate distribution and rhythmicity of hippocampal neurons. **(L)** Mean firing rates of hippocampal neurons follow a log-normal distribution, with most (73%) below 5 Hz. **(M)** Example hippocampal neurons (one per row) exhibiting 2-Hz rhythmicity. From left to right: mean spike waveform across tetrode channels; short- (millisecond) and long- (second) timescale spike autocorrelograms (ACGs); power spectral density (PSD); and inter-spike interval (ISI) distribution. Note that both hippocampal neurons with broad and narrow spike waveforms could exhibit 2-Hz rhythmicity.

(N-P) Both putative hippocampal pyramidal neurons (pPYR) and interneurons (pINT) were coupled to hippocampal 2-Hz phase. **(N)** Using peak amplitude asymmetry (b - a / b + a) and trough-to-peak latency (c) measured on the spike waveforms of individual neurons (left), pPYR (broad spikes) and pINT (narrow spikes) were separated (right). **(O)** Firing-phase histograms from two example subjects showing pPYR and pINT cells with comparable 2-Hz modulation depth, and either similar (left) or different (right) preferred phases. **(P)** Median differences in spike–phase consistency at 2 Hz between pPYR and pINT cells.

Data were analyzed using two-sided paired permutation tests except in **I** where unpaired tests were applied; ***P < 0.001, *P < 0.05; n.s., not significant.

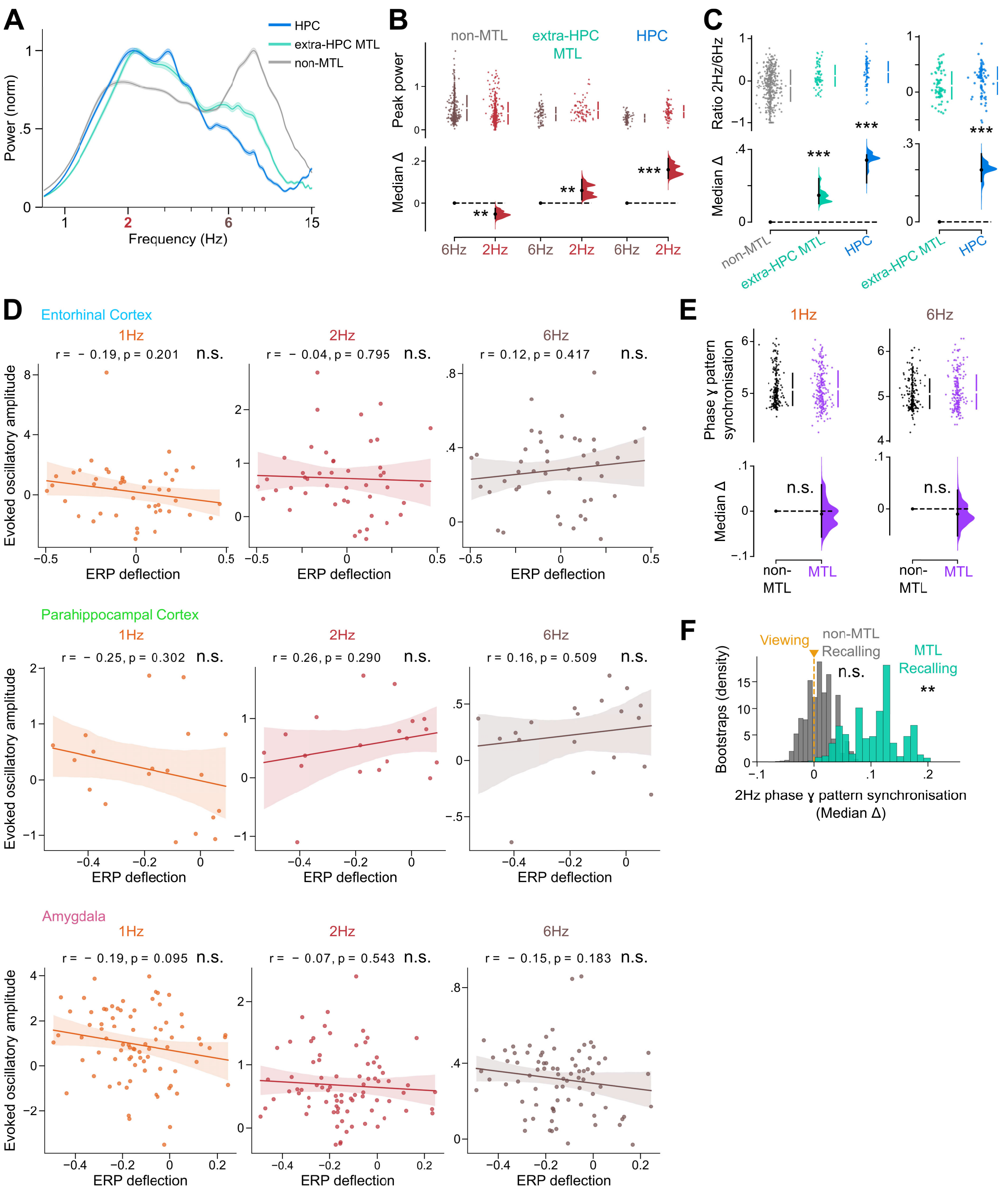

Figure S3. Oscillatory power and gamma-band synchronization across the temporal lobe. *Related to Figure 3*

(A-C) Slow-frequency oscillations across the temporal lobe. **(A)** Power spectral densities (PSDs) corrected for the aperiodic (1/f) component and averaged across hippocampal (HPC), extra-hippocampal medial temporal lobe (extra-HPC MTL), and non-MTL temporal lobe macrocontacts. All contacts were free of interictal discharges. Shaded areas indicate mean ± SEM. **(B)** Estimation plots showing mean differences between corrected 2-Hz and 6-Hz peak power in the regions shown in **A**. **(C)** Estimation plots showing higher 2-Hz/6-Hz power ratios in hippocampal and extra-hippocampal MTL contacts compared with non-MTL regions (left), and higher 2-/6-Hz power ratios in hippocampal contacts compared with extra-hippocampal MTL contacts (right).

(D) ERP deflection does not correlate with evoked 2-Hz bursts outside the hippocampus. Correlations between ERP deflection and evoked 1-, 2-, and 6-Hz amplitudes during recall in the entorhinal cortex (top), parahippocampal cortex (middle) and amygdala (bottom). No correlation reached significance.

(E,F) Cross-regional 2-Hz phase synchronization of gamma-band activity. **(E)** Estimation plot showing median differences in 1-Hz (left) and 6-Hz (right) phase synchronization between MTL and non-MTL gamma-band activity patterns during learning. **(F)** Estimation plot showing median differences in 2-Hz phase synchronization between MTL and non-MTL gamma-band activity patterns during recall relative to viewing.

Data were analyzed using two-sided paired permutation tests, except in **C** where unpaired tests were applied; ***$P < 0.001$, **$P < 0.01$; n.s., not significant.

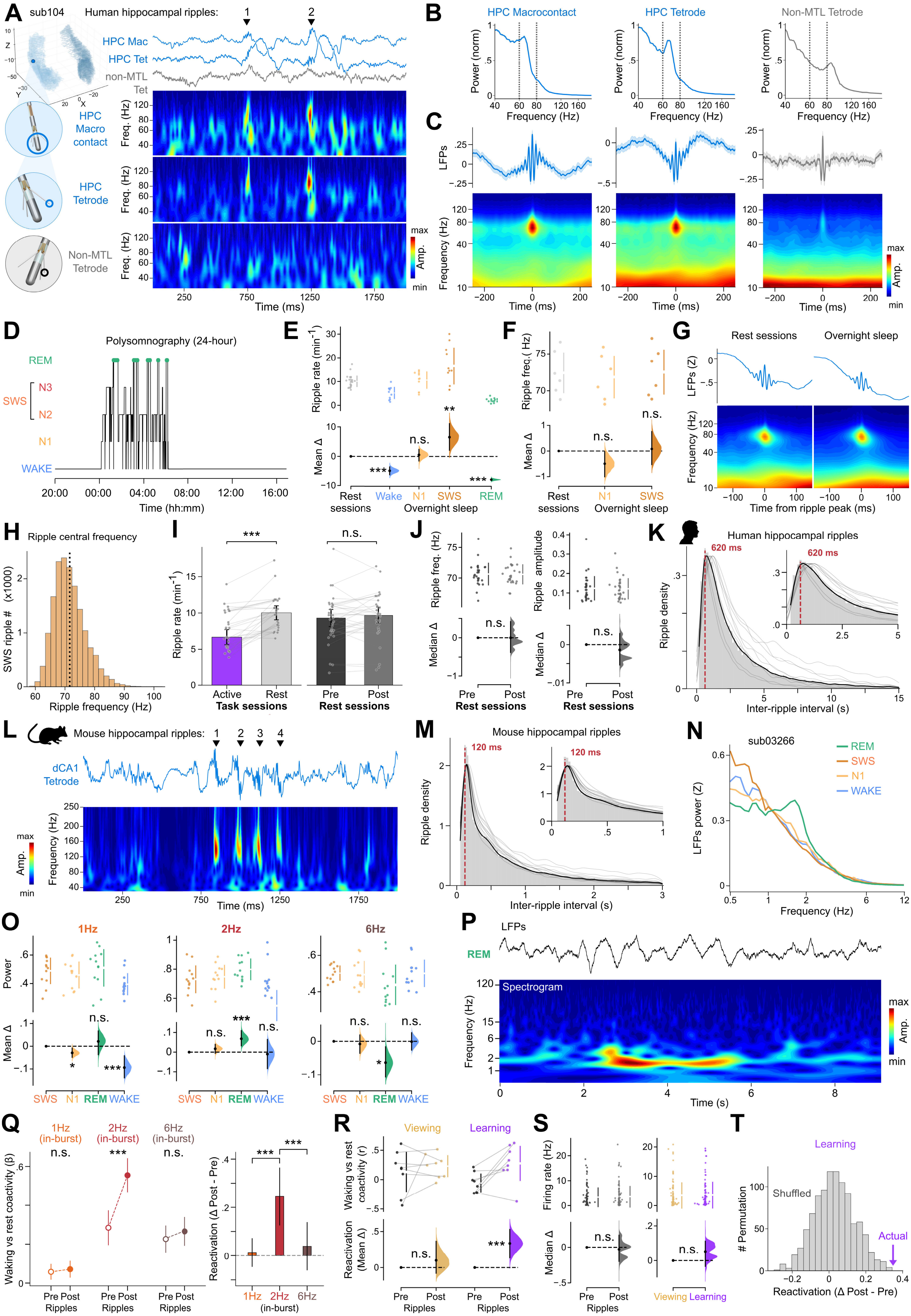
A
sub104
Human hippocampal ripples:
1
2
HPC Mac
HPC Tet
non-MTL
Tet
HPC Macro contact
HPC Tetrode
Non-MTL Tetrode
Freq. (Hz)
max
Amp.
min
Time (ms)
B
HPC Macrocontact
HPC Tetrode
Non-MTL Tetrode
Power (norm)
Frequency (Hz)
C
LFPs
Frequency (Hz)
Time (ms)
D
Polysomnography (24-hour)
REM
SWS
N3
N2
N1
WAKE
Time (hh:mm)
E
Ripple rate (min^{-1})
Mean Δ
Rest sessions
Wake
N1
SWS
REM
Overnight sleep
F
Ripple freq. (Hz)
Mean Δ
n.s.
Rest sessions
N1
SWS
Overnight sleep
G
Rest sessions
Overnight sleep
LFPs (Z)
Frequency (Hz)
Time from ripple peak (ms)
H
Ripple central frequency
SWS ripple # (x1000)
Ripple frequency (Hz)
I
Ripple rate (min^{-1})
Active
Rest
Task sessions
Pre
Post
Rest sessions
J
Ripple freq. (Hz)
Median Δ
Ripple amplitude
Pre
Post
Rest sessions
K
Human hippocampal ripples
620 ms
Ripple density
Inter-ripple interval (s)
L
Mouse hippocampal ripples:
dCA1 Tetrode
Frequency (Hz)
Time (ms)
M
Mouse hippocampal ripples
120 ms
Ripple density
Inter-ripple interval (s)
N
sub03266
REM
SWS
N1
WAKE
LFPs power (Z)
Frequency (Hz)
O
1Hz
2Hz
6Hz
Power
Mean Δ
SWS
N1
REM
WAKE
P
LFPs
REM
Spectrogram
Frequency (Hz)
Time (s)
Q
1Hz (in-burst)
2Hz (in-burst)
6Hz (in-burst)
Waking vs rest coactivity (β)
Pre Post Ripples
Reactivation (Δ Post - Pre)
1Hz
2Hz
6Hz
(in-burst)
R
Viewing
Learning
Waking vs rest coactivity (r)
Reactivation (Mean Δ)
Pre Post Ripples
S
Firing rate (Hz)
Median Δ
Pre Post Ripples
Viewing Learning
T
Learning
Shuffled
Permutation
Actual
Reactivation (Δ Post - Pre)

Figure S4. Detection, validation, and control analyses of human hippocampal ripples.
Related to Figure 4

(A-C) Ripples detected on hippocampal macrocontacts and tetrodes. **(A)** Example rest recording showing LFP traces and corresponding spectrograms from a hybrid hippocampal electrode (macrocontact and local tetrode) and from a distal, non-MTL tetrode. **(B)** Average PSDs across pre- and post-learning rest sessions showing a peak at ~70 Hz on the hippocampal macrocontact and tetrode. The distal, non-MTL tetrode shows a peak at ~85 Hz. **(C)** Ripple-triggered average LFPs and spectrograms aligned to the local peak of the macrocontact or tetrode signals, showing ripples detected on hippocampal macrocontacts and local tetrodes but not on distal, non-MTL tetrodes.

(D-J) Validation of hippocampal ripples detected during rest task sessions. **(D)** Sleep stages across a 24-h recording session in an example participant. *REM*, rapid eye movement; *SWS*, slow-wave sleep. **(E)** Estimation plot showing mean differences in ripple rate between rest sessions of the memory task and overnight sleep stages. **(F)** Estimation plot showing mean differences in ripple central frequency between rest task sessions and overnight N1 or slow-wave sleep (SWS) sessions, using the same contacts from the same subjects (n = 6 participants). Both the occurrence rate [mean (95% CI): 0.17 (0.15–0.18) Hz] and the central frequency [mean (95% CI): 70.60 (69.92–71.28) Hz] of ripples in rest task sessions were comparable to those recorded during overnight SWS. **(G)** Average LFP traces (top) and spectrograms (bottom) triggered by hippocampal ripples during rest task sessions or overnight sleep (N1), recorded from the same macrocontact in the same participant. **(H)** Distribution of ripple central frequencies across detected SWS ripples, pooled across participants recorded overnight (as in **F**). The detection algorithm identified ripples up to ~90 Hz; the black vertical dashed line indicates the median frequency (70.9 Hz; mean = 71.6 Hz). **(I)** Bar plots showing higher ripple rates during rest (pre- and post-learning) than during waking task sessions (viewing, learning, recall), with no difference between pre- and post-learning rest. **(J)** Estimation plots showing comparable ripple central frequency (left) and amplitude (right) between pre- and post-learning rest. Ripple occurrence was higher in SWS than during REM sleep and wake, and higher during pre- and post-learning rest than during awake task sessions (viewing, learning, and recall).

(K-M) Inter-ripple intervals are longer in the human hippocampus. **(K)** Histogram and kernel density estimations (black curve) showing the distribution of intervals between consecutive ripples detected in the human hippocampus during overnight slow-wave sleep (SWS); grey curves indicate inter-ripple intervals statistics from individual recording nights; red dashed line indicates the peak of the distribution (~620 milliseconds). **(L)** Example 2-second tetrode recording from the mouse hippocampus (dorsal CA1) showing the LFPs and corresponding spectrogram of consecutive ripples. Note that ripple central frequency is faster in mice[2,5,6]. **(M)** Same as in **(K)** for 12 mice recorded during >90 minutes, showing that inter-ripple intervals peak at ~120 milliseconds in mice.

(N-P) Slow oscillatory activity during REM sleep. **(N)** Power spectral densities from an example subject showing prominent 2-Hz oscillations in the human hippocampus during REM sleep, but not during SWS (see also ref [7]). Note the elevated <1-Hz power during SWS. **(O)** Mean differences across recording nights confirming that 2-Hz power is specifically elevated in the hippocampus during REM sleep. **(P)** Representative example of a ~3-second (6 cycles) hippocampal 2-Hz burst during REM sleep. Note the symmetrical oscillatory cycles during REM sleep as compared to the asymmetrical cycles observed during task engagement (Figure 2A).

(Q-T) Reactivation of multi-regional MTL coactivity motifs across rhythms and control analyses. **(Q)** β coefficients from GLMs quantifying the relationship between in-burst waking events (1-, 2-, and 6-Hz) and ripple coactivity motifs in pre- and post-learning rest (left), and corresponding reactivation strength (right; post- minus pre-learning rest). Significant reactivation was observed only for 2-Hz oscillatory bursts. **(R)** Estimation plots showing matrix-level correlations between viewing- or learning-related coactivity motifs and pre- or post-learning ripples. **(S)** Estimation plots showing similar single-neuron firing rates between pre- and post-learning ripples (left) and between viewing and learning sessions (right). **(T)** Reactivation measured from coactivity motifs computed on actual versus shuffled control spike trains. For each time bin, neuron identities were permuted to disrupt pairwise correlations. Reactivation from actual spike trains exceeded the shuffled distribution.

Data were analyzed using two-sided paired permutation tests except in **E**, where unpaired tests were applied, and Wald t-tests on GLM coefficients (**Q**, left) or interaction terms (**Q** right); $^{***}P < 0.001$, $^{**}P < 0.01$; n.s., not significant.

Supplemental references list

1. Clarke-Williams, C.J., Lopes-dos-Santos, V., Lefèvre, L., Brizee, D., Causse, A.A., Rothaermel, R., Hartwich, K., Perestenko, P.V., Toth, R., McNamara, C.G., et al. (2024). Coordinating brain-distributed network activities in memory resistant to extinction. Cell *187*, 409-427.e19. https://doi.org/10.1016/j.cell.2023.12.018.
2. Lopes-dos-Santos, V., Brizee, D., and Dupret, D. (2025). Spatio-temporal organization of network activity patterns in the hippocampus. Cell Reports *44*, 115808. https://doi.org/10.1016/j.celrep.2025.115808.
3. Buzsáki, G. (2002). Theta Oscillations in the Hippocampus. Neuron *33*, 325–340. https://doi.org/10.1016/S0896-6273(02)00586-X.
4. McHugh, S.B., Lopes-dos-Santos, V., Gava, G.P., Hartwich, K., Tam, S.K.E., Bannerman, D.M., and Dupret, D. (2022). Adult-born dentate granule cells promote hippocampal population sparsity. Nature Neuroscience *25*, 1481–1491. https://doi.org/10.1038/s41593-022-01176-5.
5. Buzsáki, G. (2015). Hippocampal sharp wave-ripple: A cognitive biomarker for episodic memory and planning. Hippocampus *25*, 1073–1188. https://doi.org/10.1002/hipo.22488.
6. Castelli, M., Lopes-dos-Santos, V., Gava, G.P., Lambiotte, R., and Dupret, D. (2025). Hippocampal ripple diversity organizes neuronal reactivation dynamics in the offline brain. Neuron *113*, 4245-4262.e17. https://doi.org/10.1016/j.neuron.2025.09.012.
7. Bódizs, R., Kántor, S., Szabó, G., Szûcs, A., Erõss, L., and Halász, P. (2001). Rhythmic hippocampal slow oscillation characterizes REM sleep in humans. Hippocampus *11*, 747–753. https://doi.org/10.1002/hipo.1090.
